# Advances in Rosin-Based Chemicals: The Latest Recipes, Applications and Future Trends

**DOI:** 10.3390/molecules24091651

**Published:** 2019-04-26

**Authors:** Szymon Kugler, Paula Ossowicz, Kornelia Malarczyk-Matusiak, Ewa Wierzbicka

**Affiliations:** 1 Faculty of Chemical Engineering, West Pomeranian University of Technology in Szczecin, Pulaskiego 10, 70-322 Szczecin, Poland; paula.ossowicz@zut.edu.pl (P.O.); kornelia.malarczyk@zut.edu.pl (K.M.-M.); 2 Industrial Chemistry Research Institute, Rydygiera 8, 01-793 Warsaw, Poland; ewa.wierzbicka@ichp.pl

**Keywords:** rosin, curing agent, resin, surfactant, biocide, medicine, polymer

## Abstract

A comprehensive review of the publications about rosin-based chemicals has been compiled. Rosin, or colophony, is a natural, abundant, cheap and non-toxic raw material which can be easily modified to obtain numerous useful products, which makes it an excellent subject of innovative research, attracting growing interest in recent years. The last extensive review in this research area was published in 2008, so the current article contains the most promising, repeatable achievements in synthesis of rosin-derived chemicals, published in scientific literature from 2008 to 2018. The first part of the review includes low/medium molecule weight compounds: Especially intermediates, resins, monomers, curing agents, surfactants, medications and biocides. The second part is about macromolecules: mainly elastomers, polymers for biomedical applications, coatings, adhesives, surfactants, sorbents, organosilicons and polysaccharides. In conclusion, a critical evaluation of the publications in terms of data completeness has been carried out with an indication of the most promising directions of rosin-based chemicals development.

## 1. Introduction

**Figure 1:**
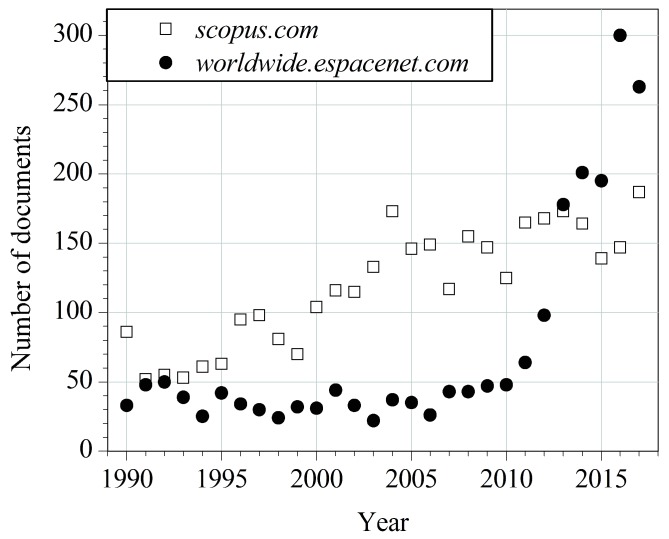
Number of scientific articles and patent documents containing the keyword “rosin” in the years 1990–2017.

The natural origin, low price, abundance and chemical modification potential of rosin make it a valuable raw material in numerous applications [1,2,3,4,5,6,7,8,9,10,11,12,13,14]. Besides the mentioned advantages, rosin is also safe for living organisms [15]. Its derivatives are claimed as non-toxic as well, despite their allergenicity [16,17]. This unique set of beneficial properties of rosin determines it as an attractive subject of innovative research characterized by a considerably growing interest in recent decades, as can be seen in Figure 1.

Unfortunately, the awareness of the possibility of using rosin as a raw material for obtaining valuable chemicals is generally unsatisfactory. There is a burning need to bring this topic to the attention of a larger group of scientists. Furthermore, the growing number of publications causes difficulties in keeping up with the latest research, as well as in selection of more promising discoveries. Sadly, the last comprehensive review document dedicated exclusively to rosin and its modifications was published in 2008 [1]. Since then, only fragmentary information on rosin has appeared in review articles on bio-based polymers and resin systems [2,3,4,5,6,7,8,9,10,11,12,13,14,15,16,17,18,19,20,21], as well as in reviews on rosin derivatives in catalysis [12], controlled drug-delivery systems [13] and small-molecule compounds [14]. 

In view of these facts, publication of a wide, comprehensive and critical review of achievements since 2008 is an important solution to solve the problem of a severe lack of current review literature in this field. The growing number of publications is not the only obstacle in creating a literature review on rosin. No less important challenge is to collect information from existing literature. Quite often articles do not contain full information about a particular reaction, but refer to earlier articles, which, in turn, may refer to even earlier articles, which may not be available online, or may not be in English. 

The current article is a direct answer to the aforementioned issues. Its aims are: (I) to provide a precise review of the scientific literature from 2008 to 2018, (II) select promising studies with clear practical application and (III) an overall assessment of the reviewed achievements in order to identify the most perspective development directions of rosin-based chemicals. The review is presented in a modern, pleasant-to-browse form, illustrated with patiently completed reaction schemes. The article provides concise, but exhaustive information on the achievements in preparation of rosin-derived chemicals in the last decade. Its main idea is to inspire and encourage the world of science to actively take interest in rosin and the possibilities of its modification.

## 2. Basic Information about Rosin

Rosin, or colophony, is a solid and brittle mixture of non-volatile conifer tree resin components. It can display colors ranging from almost colorless, through shades of yellow and brown to black. Depending on the origin, two industrially important types of rosin can be distinguished, i.e., gum rosin and tall oil rosin. Gum rosin is the non-volatile residue remaining after distillation of tree resin obtained by tapping of living trees. Tall oil rosin is a by-product of wood pulping in the kraft process. Gum rosin accounts for ca. 60% of world rosin production, whereas tall oil rosin is ca. 35%. In the past, rosin was widely obtained from the solvent extraction of harvested wood as so-called wood rosin, but nowadays, this technology is of little importance [1,22]. The price of rosin in the first half of 2018 ranged from 300 to 2750 USD/ton depending on its origin, supplier and color. Annual world production of rosin is ca. 1.2 million tons and has remained stable in the last decades [1,2,22].

**Figure 2:**
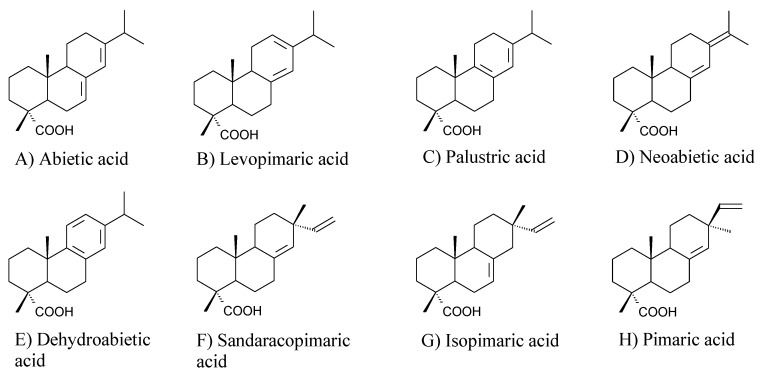
Structural formulas of the most common abietane-structured (**A**–**E**) and pimarane-structured (**F**–**H**) resin acids.

**Scheme 1:**
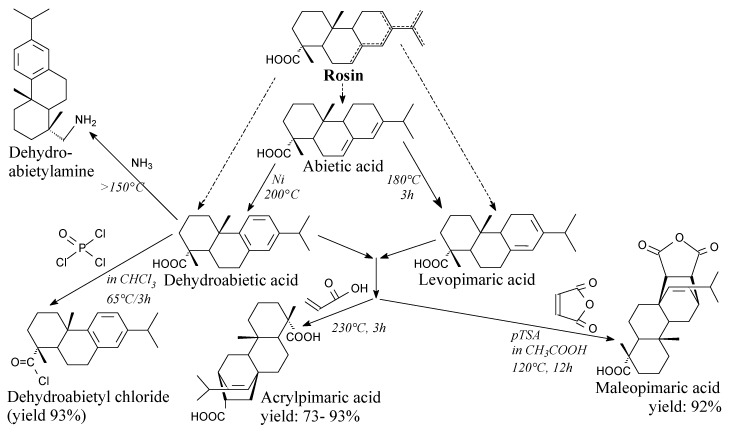
Synthesis of essential rosin-based substrates/intermediates.

Resin acids, with the general formula C_19_H_29_COOH, constitute up to 95 wt.% of rosin, while neutral compounds are present in amounts of a few percent. A number of resin acids based on a few diterpene carbon skeletons were identified so far [1]. The most abundant resin acids are built on abietane and pimarane skeletons. Their structural formulas are shown in Figure 2. It should be emphasized, that abietane-structured acids are characterized by conjugated double bond systems, which makes them particularly susceptible to chemical modifications. It is noteworthy, that abietane-structured acids isomerize at elevated temperature to afford readily reactive levopimaric acid, according to Scheme 1. On the other hand, pimarane-type acids do not have conjugated double bond systems, which limits their chemical processability. The detailed chemical composition of rosin depends on its type (gum, tall oil or wood), thermal history, species of tree and geographical origin [23,24]. Compositions of gum rosin from various sources are presented in Table 1, where it can be seen that the content of abietane-type acids may vary between 64 and 87 wt.%. Such detailed composition of a rosin can be determined, e.g., by the capillary electrophoresis method [25].

In recent studies unmodified rosin was used for making films, coatings and adhesives [26,27,28,29,30,31,32,33,34,35,36,37,38,39,40,41,42], biomedical applications [43,44,45,46,47,48,49,50,51,52,53,54,55], or in mining, metallurgy and construction [56,57,58] and for filler purposes [59,60,61,62,63,64]. However, chemical modification of rosin gives many-fold greater possibilities of using this raw material, which are described in the next, main section of this article.

## 3. Rosin-based Chemicals

### 3.1. General Comments on the Whole Review

The review contains short, but exhaustive descriptions of rosin-derived compound syntheses published in the scientific literature from the JCR list since 2008. It does not include older achievements in the field of rosin, that were widely described in previous review literature [1,65] and commercialized [66,67,68,69,70,71,72,73,74,75,76,77,78,79,80]. It includes preparations of completely new chemicals, as well as new ways to synthesize already known compounds. Only products with declared or obvious practical applications have been chosen. The review is divided into two sections according to the general molecular structure of the prepared compounds, while each section is divided according to practical application and the structure similarity of the compounds. The collected data are given in the article text and in schemes. The following information are presented in the text: product name, product morphology, substrates name(s), separation techniques and practical applications. On the other hand, data such as reaction scheme, catalyst use, reaction media, temperature, pressure, time and yield are included in the schemes. There are situations where some data simply has not been reported by original authors, e.g., product morphology or yield. Such missing data could not be presented in this review. What does the above mean? The more data provided, the more advanced the research on a compound, and the more reliable the recipe. The lack of data should indicate that the research on a certain rosin derivative is probably at an early, basic stage. It has to be underlined that reaction schemes do not take into account advanced stereochemistry. Finally, almost all reactions were conducted under an inert atmosphere: nitrogen or argon, so reaction schemes do not include this information.

### 3.2. Small and Medium Molecule Compounds

This section describes rosin-derived small/medium molecule compounds with strictly defined structures that do not contain the repeated units typical for macromolecules. 

#### 3.2.1. Intermediates

Intermediates are the products of simple rosin modifications, which are necessary for the preparation of many new compounds. They can be synthesized in very simple, well described ways with high yields. The sustainability of these processes is high: They usually use a biobased main substrate (rosin) in solvent-free processes. Some drawback can be the separation processes, that may not always be easy, because of the high viscosity, m.p. and stickiness of products. In view of the above, rosin-based intermediates have very high commercialization potential and some of them are commercially available in certain regions of the world.

Maleopimaric acid is an off-white solid (m.p. 223 °C). It can be prepared from abietic acid and maleic anhydride via a Diels-Alder reaction according to Scheme 1 [81], followed by recrystallization [82]. Maleopimaric acid is one of the crucial products in rosin chemistry. It can be used directly as an epoxy resin hardener, but its applications are much wider. They include preparation of epoxy resins [83,84], acrylic resins [85,86,87], allyl resins [88,89,90], polyols [91], bio-based curing agents for synthetic epoxy resins [92,93,94,95,96,97,98] or bio-based ones [99], surfactants [100,101,102,103], intermediates [104,105], biologically active compounds [105,106,107,108], polyurethanes [109,110,111,112], chemicals for NMR techniques [113] and photolitography [114], sorbents [115], organosilicon compounds [116], printing inks [117] and for the hydrophobization of wood surfaces [118,119,120]. It is noteworthy that the main degradation products of maleopimaric acid are water, carbon dioxide, formamide and also aliphatic and aromatic derivatives [121]. It is worth noting that fumaropimaric acid is an isomer of hydrated maleopimaric acid, which can be synthesized in a similar way [122], but its importance to rosin chemistry is much smaller: it can be used in synthesis of triglycidyl epoxy resin [122] and water-borne polyurethanes [109].

Acrylpimaric acid is an off-white solid (m.p. 220 °C). It can be prepared from abietic acid and acrylic acid according to Scheme 1, followed by precipitation, filtration, washing and recrystallization [123,124]. It is noteworthy that neat rosin can be acrylated as well [125]. As an important material in rosin chemistry, acrylpimaric acid can be used for preparation of diallyl acrylpimarate [123], polyesters of acrylated rosin and polyethylene glycols [125], epoxy resins [126,127], acrylpimaryl dichloride [128,129], acrylpimaric acid amides [130], cyclic diamide [131], quaternary ammonium salts [132,133,134,135,136], calcium and zinc salts [137] as well as polyesters [138]. Moreover, it can be used directly as an epoxy curing agent [139].

Another important compound in this subsection is dehydroabietyl chloride. It is a viscous, yellow, oily liquid [140,141]. It can be prepared using: (i) oxalyl chloride according to Scheme 1 prior to evaporation of unnecessary substances [142], (ii) phosphorus trichloride in chloroform (yield 92.5%) [140] or (iii) thionyl chloride in presence of 4-dimethylaminopyridine [143]. It can be used as a substrate for the synthesis of macroinitiators for atom transfer radical polymerization (ATRP) reactions [142], rosin phosphate esters [140], *N*-hydroxyethylacrylamide ester of dehydroabietic acid [144], dehydroabietic ethyl methacrylate [145,146], dehydroabietic propargyl ester [147] dehydroabietic hexyl acrylate [148], as well as other intermediates in synthesis of various surfactants [143,149] and medicines [141,150,151].

Dehydroabietylamine, also known as leelamine, is a solid (m.p. 44.5 °C). It is commercially available. Its application in antitumor therapies was investigated in recent years [152,153,154,155]. Moreover, it can be a substrate for preparation of epoxy resin [156], bio-based benzoxazines [157], quaternary ammonium surfactants [158,159], acrylic monomers: glycidyl methacrylate monomer [160] or *N*-dehydroabietic acrylamide [161].

#### 3.2.2. Resins and Monomers

Rosin-based resins and monomers are compounds which contain epoxy, acrylic, allyl, hydroxyl or oxazine reactive groups, that enable cross-linking, polymerization or building in the polymer matrix. Their preparations are usually well described and easy to perform. The syntheses are similar to conventional resins/monomers preparations, however the necessity of using organic solvents in several reactions is a disadvantage in the context of Green Chemistry rules. The high modification potential of rosin derivatives allows one to prepare resins and monomers showing diverse and designable properties. They can exhibit adjustable glass transition temperatures, low volume shrinkage, as well as improve elastic modulus, Young’s modulus, shape-memory, flame retardancy, corrosion protection features of final casts/polymers in comparison with petroleum-based compounds. Therefore, the best described recipes show high commercialization potential in the segment of resins, adhesives and paints, but in most cases, additional applied research should be performed to increase their technology readiness level (TRL).

**Scheme 2:**
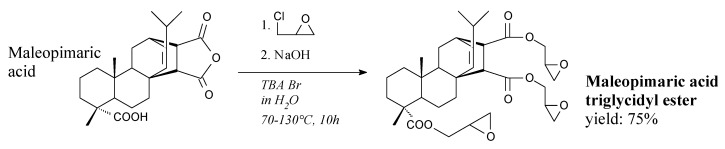
Synthesis of triglycidyl ester of maleopimaric acid.

Triglycidyl ester of maleopimaric acid is a beige, viscous liquid. It can be prepared from maleopimaric acid, epichlorohydrin and sodium hydroxide according to Scheme 2, followed by filtration, washing and evaporation [83]. It can be used in liquid epoxy resins [83], non-cytotoxic bio-based epoxypolyurethanes [162] as well as synthesis of rosin-based cyclic carbonates [132]. Similar liquid resin can be synthesized from fumaropimaric acid [122].

**Scheme 3:**
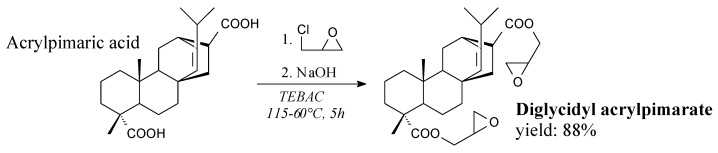
Synthesis of diglycidyl acrylpimarate.

Diglycidyl acrylpimarate is a yellowish liquid. It can be prepared from acrylpimaric acid, epichlorohydrin and sodium hydroxide according to Scheme 3, prior to filtration, washing and drying [126]. It can be used in epoxy materials showing improved thermal, mechanical and shape-memory properties [126].

**Scheme 4:**
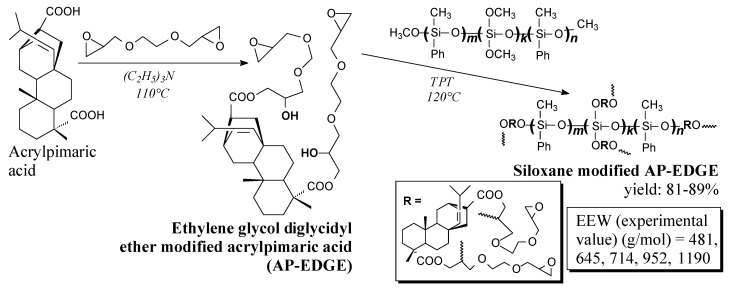
Preparation of ethylene glycol diglycidyl ether modified acrylpimaric acid and its siloxane derivative.

Another diglycidyl derivative of acrylpimaric acid and its siloxane modification can be prepared according to Scheme 4, prior to washing, filtration and vacuum evaporation [127]. Prepared epoxy resins can improve the thermal stability and flame retardancy of products [127].

**Scheme 5:**
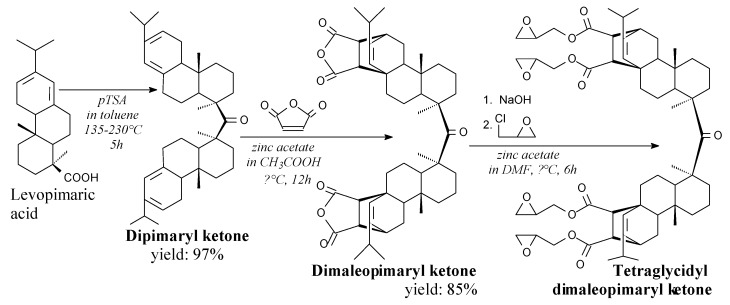
Synthesis of tetraglycidyl dimaleopimaryl ketone.

Dimaleopimaryl ketone is a brownish yellow solid. It can be prepared from levopimaric acid and maleic anhydride according to Scheme 5, prior to washing and recrystallization [163]. It can be used directly as an epoxy resin hardener, as well as for the synthesis of bio-based epoxy resins, i.e., tetraglycidyl dimaleopimaryl ketone, according to Scheme 5 [163].

**Scheme 6:**
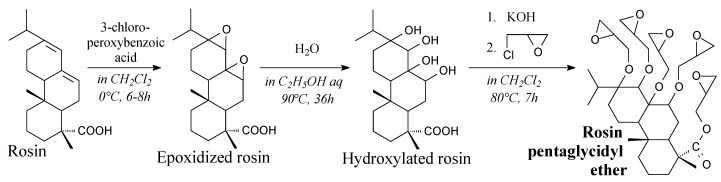
Synthesis of rosin pentaglycidyl ether.

Rosin pentaglycidyl ether is a solid. It can be prepared from epoxidized rosin, water, potassium hydroxide and epichlorohydrin according to Scheme 6, and using such separation methods as vacuum drying, filtration, precipitation and washing [164]. It can be used as a component in epoxy resin systems showing high glass transition temperature as well as elastic modulus [164].

**Scheme 7:**
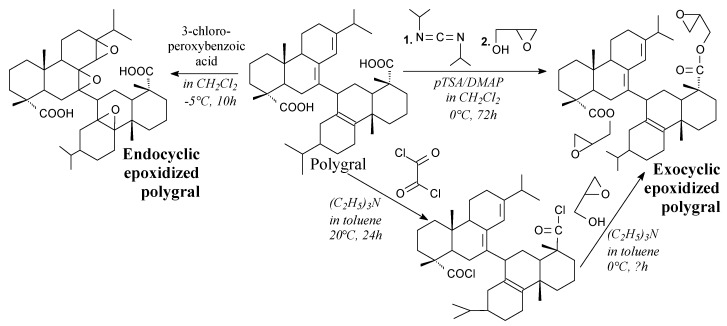
Preparation of epoxidized rosin oligomers.

Polygral is a solid byproduct of the paper and forestry industry, containing rosin acids and their oligomers [165]. It can be epoxidized to prepare bio-based epoxy resins. Endocyclic epoxidized polygral is a red brown solid, which can be prepared using 3-chloroperoxybenzoic acid, according to Scheme 7, prior to vacuum evaporation. On the other hand, exocyclic epoxidized polygral is a viscous red brown liquid, that can be prepared in two ways, using oxalyl chloride or *N*,*N*′-diisopropyl carbodiimide before addition of glycidol, according to Scheme 7 prior to washing and vacuum drying. They can be used for preparing bisphenol A-free epoxy resins [165].

**Scheme 8:**
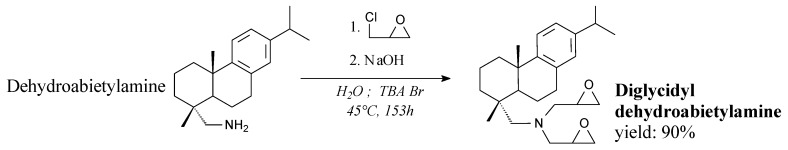
Synthesis of diglycidyl dehydroabietylamine.

Diglycidyl dehydroabietylamine is a yellowish sticky liquid. It can be synthesized from dehydroabietylamine, epichlorohydrin and sodium hydroxide according to Scheme 8, prior to filtration, washing, drying and vacuum distillation [156]. It can be applied in epoxy resins exhibiting better thermal stability and higher glass transition temperatures than petroleum-based products [156].

**Scheme 9:**
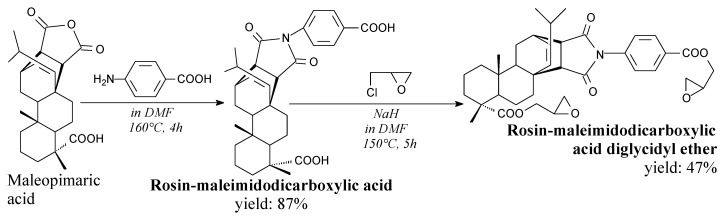
Preparation of rosin maleimidodicarboxylic acid diglycidyl ether.

Rosin maleimidodicarboxylic acid diglycidyl ether is a yellowish solid. It can be prepared from epichlorohydrin and dicarboxylic derivative of maleimide (described in more detail in Section 3.2.3) according to Scheme 9 prior to filtration, washing, drying and rotary evaporation [84]. It shows higher glass transition temperature, modulus and thermal stability than its plant oil counterparts [84].

**Scheme 10:**
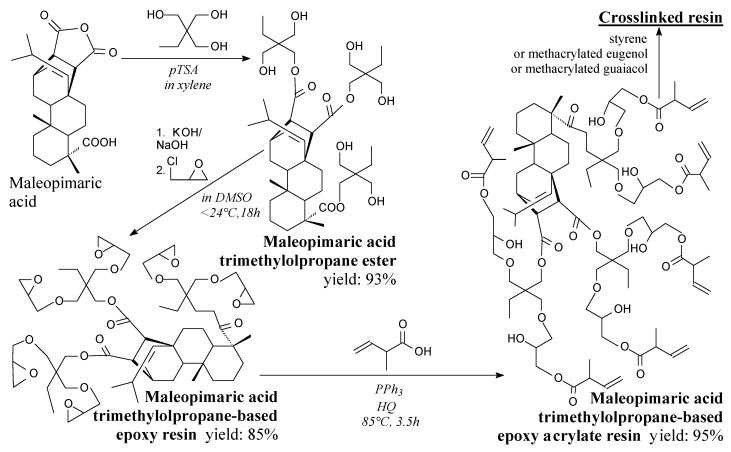
Preparation of maleopimaric acid-trimethylolpropane ester-based epoxy acrylate resin crosslinked by unsaturated monomers.

Triester of maleopimaric acid and trimethylolpropane is a dark yellow solid. It can be prepared according to Scheme 10, prior to washing and vacuum distillation [98]. It can be used in synthesis of rosin-based epoxy resin [98].

Epoxy resin having EEW = 199.68 g/eq based on maleopimaric acid and trimethylolpropane ester can be prepared according to Scheme 10, prior to extraction and vacuum distillation [98]. It can be used directly as an epoxy compound, or transformed into acrylate resin [98].

Acrylate resin based on epoxidized maleopimaric-trimethylolpropane adduct can be prepared according to Scheme 10 [98]. It can be used as a resin crosslinked by styrene, methacrylated eugenol or methacrylated guaiacol [98].

**Scheme 11:**
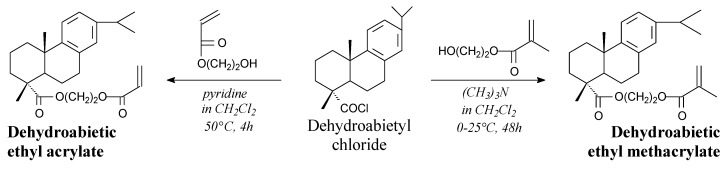
Synthesis of dehydroabietic ethyl acrylates.

Dehydroabietic ethyl methacrylate is a white powder. It can be prepared from dehydroabietyl chloride and hydroxyethyl methacrylate according to Scheme 11, prior to neutralization, drying, evaporation and chromatography [146]. It can be widely used as a monomer in preparation of graft and block copolymers [146,166,167,168,169,170]. It is worth noting, that a similar compound dehydroabietic ethyl acrylate is a yellow, viscous liquid, which can be prepared from hydroxyethyl acrylate and dehydroabietyl chloride prior to filtration, precipitation and vacuum distillation [171]. It can be used for preparation of homopolymer with no declared application [171]. Moreover, rosin ethyl acrylate can be used in preparation of bio-based graft copolymers of chitosan for controlled release applications [172].

**Scheme 12:**
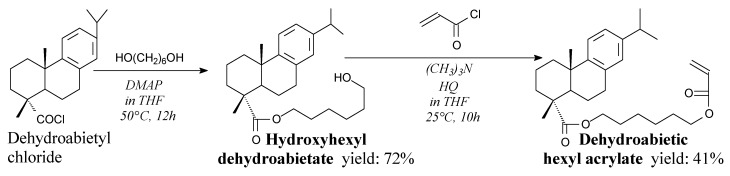
Synthesis of dehydroabietic hexyl acrylate.

Dehydroabietic hexyl acrylate is a solid. It can be synthesized from dehydroabietyl chloride, hexanediol and acryloyl chloride according to Scheme 12, and using separation methods such as filtration, precipitation, washing and vacuum drying [148]. Its application is a soft acrylic monomer (glass transition temperature of −23 °C), which can impart a flexibility to the integrated polymer [148].

**Scheme 13:**
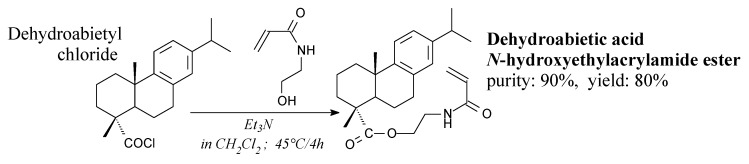
Synthesis of *N*-hydroxyethylacrylamide ester of dehydroabietic acid.

*N*-hydroxyethylacrylamide ester of dehydroabietic acid is a viscous, yellow liquid. It can be prepared from dehydroabietyl chloride and *N*-hydroxyethylacrylamide according to Scheme 13, prior to filtration, washing, drying and column chromatography [144]. It can be used as a monomer in thermoset system with soybean oil-based resin for coating and adhesive applications [144].

**Scheme 14:**
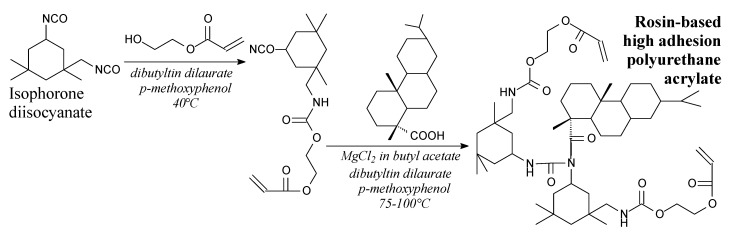
Synthesis of rosin-based high adhesion polyurethane acrylate.

Rosin-based high adhesion polyurethane acrylate is a faint-yellow solid. It can be synthesized from hydrogenated rosin, isophorone diisocyanate and 2-hydroxyethyl acrylate according to Scheme 14, prior to precipitation, vacuum drying and column chromatography [173]. Its application is an adhesive having a high polymerization rate, low volume shrinkage and high adhesion [173].

**Scheme 15:**
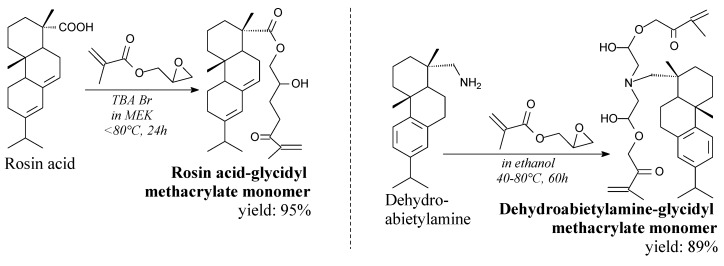
Synthesis of rosin-based glycidyl methacrylate monomers.

Rosin-based glycidyl methacrylate monomers are viscous liquids: brown rosin acid-glycidyl methacrylate and colorless dehydroabietylamine-glycidyl methacrylate. They can be prepared from glycidyl methacrylate and rosin or dehydroabietylamine according to Scheme 15, prior to the use of such separation techniques as washing, extraction and evaporation [160,174]. They significantly improve thermal and mechanical properties of soybean oil-based thermosets [160]. Moreover, they can be used in copolymerization with other acrylate monomers [175], or as an advanced tackifier in the UV-crosslinking pressure sensitive adhesives [174,176].

**Scheme 16:**
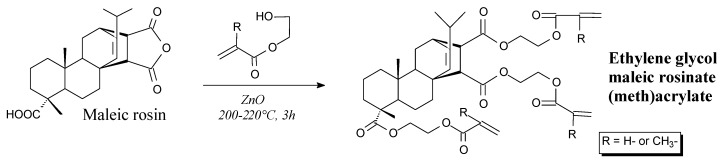
Preparation of ethylene glycol maleic rosinate acrylate.

Ethylene glycol maleic rosinate (meth)acrylate can be prepared from maleated rosin, ethylene glycol and (meth)acrylic acid according to Scheme 16 [85]. It can be applied in styrene-acrylate copolymers increasing their thermal stability [86], as well as in preparation of moleculary imprinted polymers for stationary phases used in high-performance liquid chromatography [85,87].

**Scheme 17:**
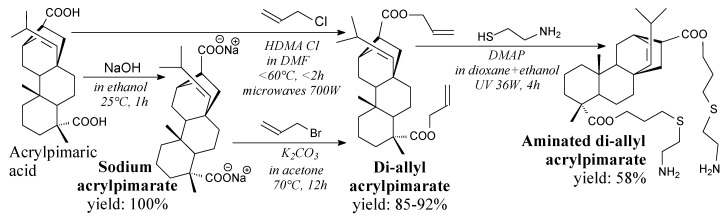
Synthesis of diallyl acrylpimarate and its aminated derivative.

Diallyl acrylpimarate is a yellow liquid [123]. It can be prepared from acrylpimaric acid and an allyl halide by different methods, according to Scheme 17, and using separation methods such as filtration, washing, extraction and evaporation [88,123,177]. It can be applied as a monomer in polyester unsaturated resins from renewable resources [123], or in synthesis of aminated curing agent for epoxy [178].

Aminated diallyl acrylpimarate is a yellow-brown solid. It can be synthesized from diallyl acrylpimarate and cysteamine hydrochloride according to Scheme 17, prior to washing, extraction and evaporation [178]. It can be applied as a resin or a curing agent for epoxy resins, giving them improved thermal and shape-memory properties [178].

**Scheme 18:**
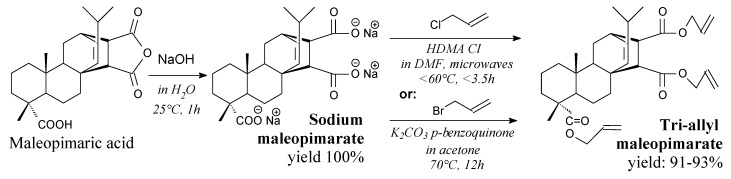
Synthesis of triallyl maleopimarate.

Sodium maleopimarate is a solid, which can be prepared from maleopimaric acid according to Scheme 18 prior to drying at 40 °C [89]. Its applications include the synthesis of triallyl maleopimarate. 

In a similar way dehydroabietic acid salts can be synthesized for use as nucleating agents for isotactic polypropylene in a non-isothermal crystallization process, improving the crystallization temperature and accelerating the nucleation rate [179,180]. Triallyl maleopimarate is a white viscous liquid, which can be prepared from sodium maleopimarate and allyl halide according to Scheme 18, prior to extraction, washing, filtration and vacuum distillation [88,89]. It can be used as a monomer in UV-cured polymer films and coatings, giving them improved adhesion and mechanical properties [89], as well as in thermally cured fully bio-based resin systems exhibiting satisfactory thermal and mechanical properties [88].

**Scheme 19:**
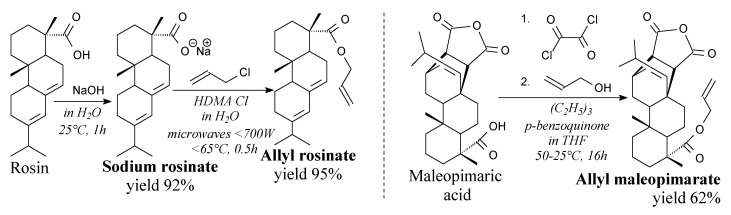
Synthesis of mono-allyl rosin derivatives.

Mono-allyl rosin derivatives have been also synthesized in recent years. Allyl rosinate can be prepared in aqueous [181] or ethanol [182] medium from rosin, sodium hydroxide and allyl chloride according to Scheme 19, prior to filtration and distillation [181]. It can be potentially applied as an unsaturated monomer in copolymerization reactions [181] as well as in UV-cured resins [182]. On the other hand, allyl maleopimarate can be prepared in THF from maleopimaric acid, oxalyl chloride and allyl alcohol according to Scheme 19, and using such separation methods as vacuum distillation and column chromatography [90]. It can be used in unsaturated resins and epoxy resins as a cross-linking agent [90].

**Scheme 20:**
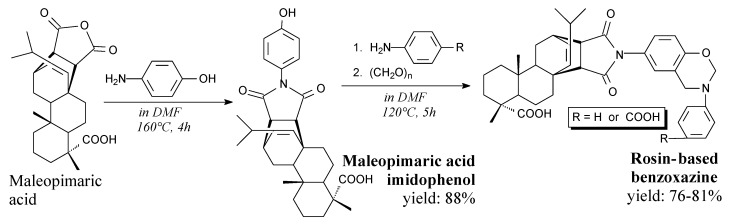
Synthesis of maleopimaric acid imidophenol and its benzoxazines.

Benzoxazines are compounds consisting of a bicyclic group with an oxazine moiety. Maleopimaric acid imidophenol is a white, crystal solid. It can be synthesized from maleopimaric acid and 4-aminobenzoic phenol according to Scheme 20, prior to precipitation and drying [96]. It can be used in synthesis of benzoxazine monomers [96].

Rosin-based benzoxazine monomers are orange solids. They can be synthesized from maleopimaric acid, paraformaldehyde and aniline or 4-aminobenzoic acid, according to Scheme 20, prior to filtration, washing and rotary evaporation [96]. They can be polymerized into products of significant thermal stability [96].

**Scheme 21:**
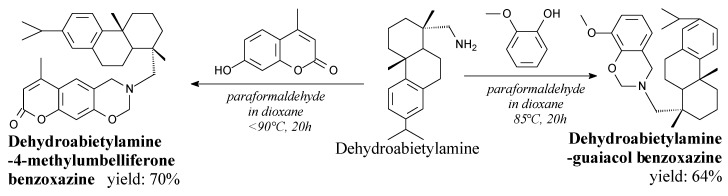
Synthesis of fully bio-based benzoxazines.

Dehydroabietylamine-guaiacol (brown powder, m.p. 104 °C) and dehydroabietylamine-4-methylumbelliferone (yellow spherical crystal solid, m.p. 131 °C) are fully bio-based benzoxazines [157]. They can be synthesized from dehydroabietylamine via Mannich condensation, according to Scheme 21. They can compound resins with strong corrosion protection and thermal stability [157].

#### 3.2.3. Hardeners

Rosin-based hardeners, i.e., curing agents, are compounds containing anhydride, carboxyl or hydroxyl groups, which allows one to apply them in epoxy or urethane resin systems. In comparison with petroleum-based hardeners they bring improved endurance and thermal properties to resin systems. Moreover, rosin-based anhydrides are non-toxic, as opposed to conventional anhydride curing agents. Their preparation is simple and well described, and usually does not require the use of solvents. These strengths cause the great interest in use of rosin based chemicals as hardeners of conventional and bio-based resins resulting in several commercializations of e.g., maleated rosins.

**Scheme 22:**
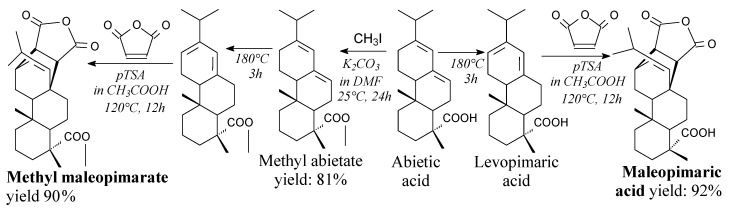
Synthesis of maleopimaric acid and methyl maleopimarate.

Methyl maleopimarate is a white solid. It can be prepared from abietic acid, iodomethane and maleic anhydride, according to Scheme 22, prior to recrystallization [82]. It can be used as bio-based curing agent for epoxies [82,183], as well as in synthesis of binaphthyl-appended crown ethers derived from rosin [184].

**Scheme 23:**
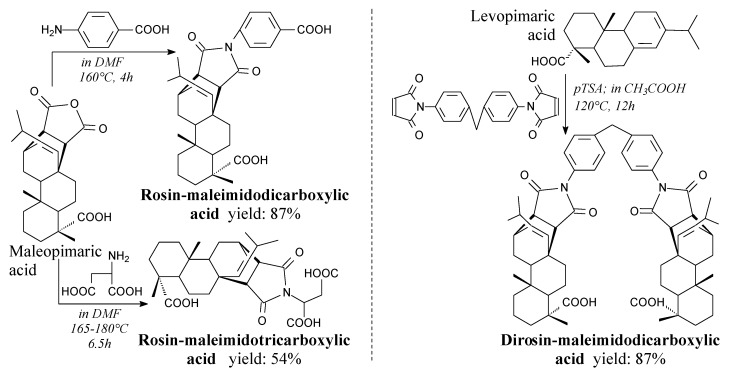
Synthesis of rosin-maleimidodicarboxylic acids.

Rosin-maleimidopolycarboxylic acids are white/yellowy or gray/brown powders. They can be prepared from maleopimaric acid with aspartic or 4-aminobenzoic acid, or rosin with 1,1′-(methylenedi-4,1-phenylene)bismaleimide, according to Scheme 23, before separation via such methods as precipitation, filtration, washing, drying and recrystallization [94,185]. They have a potential to replace petroleum-based epoxy curing agents [94,185]. Furthermore, rosin-maleimidodicarboxylic acid can be used for synthesis of rosin-based chain extender for polyurethanes [186], or epoxy resins [84].

**Scheme 24:**
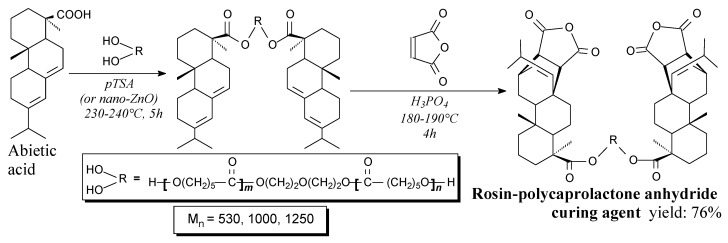
Synthesis of rosin-polycaprolactone anhydride curing agent.

Rosin-polycaprolactone flexible dianhydride are solids with a melting point depending on the length of oligoester diol chain. They can be synthesized from rosin, oligocaprolactone diols and maleic anhydride according to Scheme 24 and using vacuum evaporation as a separation method [97]. Their application is bio-based curing agent for epoxy resins [97].

**Scheme 25:**
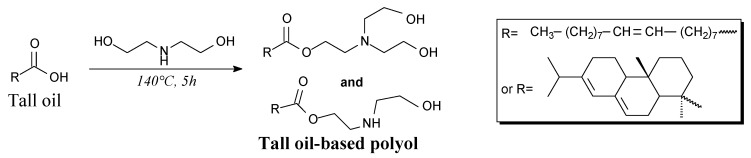
Synthesis of tall oil-based polyol.

Tall-oil based polyol can be prepared from diethanolamine and tall oil containing up to 20 wt.% of rosin acids, according to Scheme 25, prior to vacuum evaporation of water [187]. It can be used as chain extenders in ureaurethane elastomers, improving their thermal resistance and storage moduls [187].

#### 3.2.4. Surfactants

Small/medium molecule rosin-based surfactants are ionic or non-ionic compounds that lower surface tension between different substances. In comparison with petrochemical counterparts, they are characterized by designable surface activity, affinity to many chemicals (especially cycloaliphatic and aromatic), non-toxicity, mild biocidal properties and enhanced thermal stability. Unfortunately, their syntheses are less well described than rosin-based resins and hardeners, e.g., reaction yields are often unavailable. Moreover, the use of unsustainable chemicals in the mentioned reactions noticeably decreases the “green” aspect of these rosin derivatives. Therefore, it is an urgent need to undertake applied studies on these compounds, as well as to find more sustainable preparation processes in order to increase TRL of this group of rosin derivatives.

**Scheme 26:**
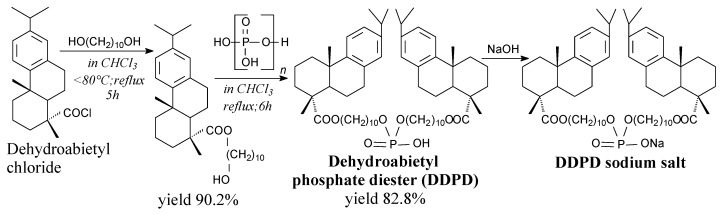
Synthesis of dehydroabietyl phosphate diester sodium salt.

Dehydroabietyl phosphate diester is a yellow viscous liquid. It can be prepared from dehydroabietyl chloride, decanediol, and polyphosphorus acid according to Scheme 26, prior to washing, vacuum evaporation and drying [140]. It can be used as highly-active surfactant [140], phosphorus source and crystal growth control agent in synthesis of hydroxyapatite [188].

**Scheme 27:**
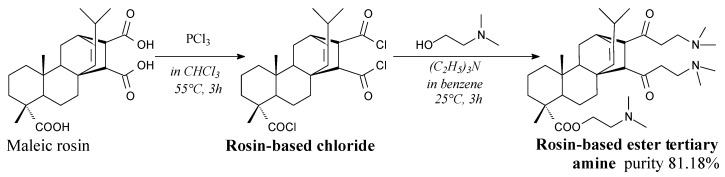
Synthesis of rosin-based ester tertiary amine.

Rosin-based chloride can be prepared from maleated rosin and phosphorus trichloride according to Scheme 27, prior to evaporation [101]. It can be used as a substrate for synthesis of rosin-based ester amines [101].

Rosin-based ester tertiary amine can be prepared from rosin chloride and *N,N*-dimethyl ethanolamine according to Scheme 27, prior to washing, drying, extraction, evaporation and recrystallization [101]. Potential applications include drug carriers and surfactants. Its surface activity significantly increases in presence of rosin phosphate ester (Scheme 26) [101].

**Scheme 28:**
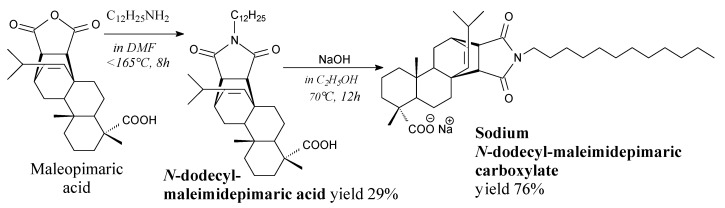
Synthesis of sodium N-dodecyl-maleimidepimaric carboxylate.

Solid *N*-dodecyl-maleimidepimaric acid (C12-MPA) can be prepared from maleopimaric acid and dodecylamine according to Scheme 28, prior to washing, drying, filtration, rotary evaporation and purification via column chromatography [100]. Sodium *N*-dodecylmaleimidepimaric carboxylate is a product of C12-MPA neutralization using NaOH, prior to evaporation, recrystallization and vacuum drying. It forms micelles of various shapes, depending on its concentration [102]. It can be used in oil extraction, cosmetics and industrial washing [100].

**Scheme 29:**
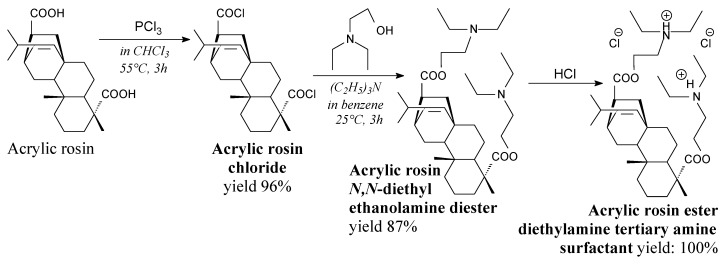
Preparation of acrylic rosin ester diethylamine tertiary amine surfactant.

Acrylpimaryl dichloride is an orange, sticky paste. It can be prepared from acrylpimaric acid according to Scheme 29, prior to solvent evaporation [128]. It can be used in synthesis of surfactants [128], herbicides [129], fungicides [189] and insecticides [190]

Acrylic rosin ester diethylamine tertiary amine surfactant is a sticky paste. It can be prepared from acrylpimaryl chloride, *N,N*-diethylethanolamine and hydrochloric acid according to Scheme 29 [128]. This product, in a mixture with soapnut saponin exhibits noteworthy surface activity and emulsification ability to apply in pharmacy, cosmetics and commodity chemicals [128].

**Scheme 30:**
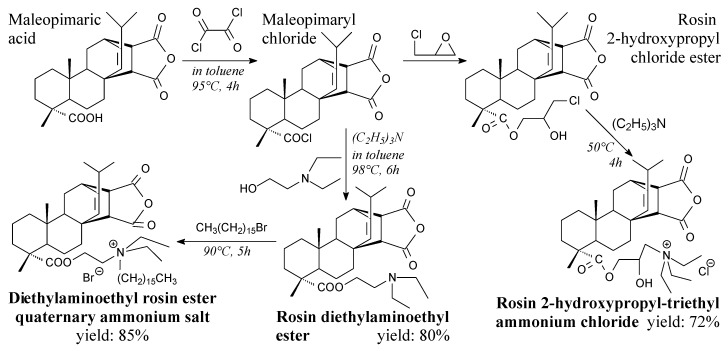
Synthesis of quaternary ammonium salts of rosin esters.

Quaternary ammonium salts of rosin esters are yellow solids. They can be prepared from maleopimaryl chloride (described in Section 3.2.5) and *N,N*-diethylethanolamine with hexadecyl bromide, or epichlorohydrin with triethylamine, according to Scheme 30, and using such separation methods as vacuum distillation, filtration, washing, drying, extraction and recrystallization [104,191]. 

Their potential applications include corrosion inhibitiors [104,191,192] dispersants for magnetite (Fe_3_O_4_) nanoparticles [191,192] and inhibitors in protein aggregation processes [193,194,195]. In addition, a similar rosinyl triquaternary ammonium salt having antifungal activity was also reported [196].

**Scheme 31:**
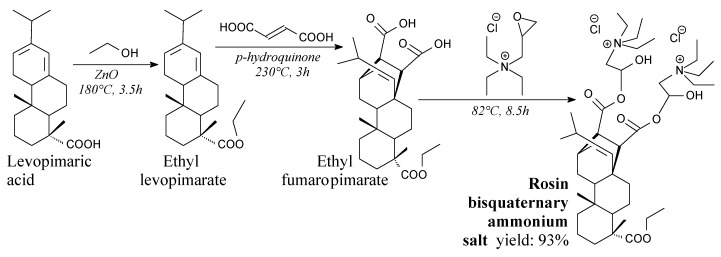
Preparation of rosin bisquaternary ammonium salt.

Another approach to introduce quaternary ammonium moieties into rosin is presented in Scheme 31 [197]. As it can be seen, rosin bisquaternary ammonium chloride can be prepared from rosin, ethanol, fumaric acid and epoxy quaternary ammonium salt, and using such separation methods as washing and drying. Thus obtained gemini surfactant has good surface activity and antifungal activity against fungi responsible for wood decay [197].

**Scheme 32:**
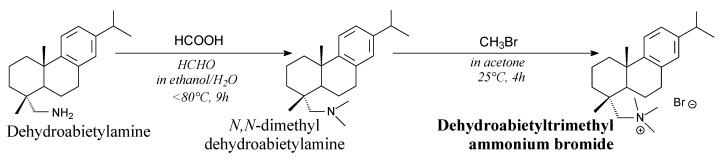
Synthesis of dehydroabietyltrimethyl ammonium bromide.

Dehydroabietyltrimethyl ammonium bromide is a yellow solid. It can be prepared from dehydroabietylamine, formic acid and methyl bromide according to Scheme 32, and using such separation techniques as washing, extraction and evaporation, drying, vacuum distillation and recrystallization [158]. Such surfactant can be used for preparation of ordered porous titania [159], zirconia [198] or silica [158] with potential applications in catalysis and separation.

**Scheme 33:**
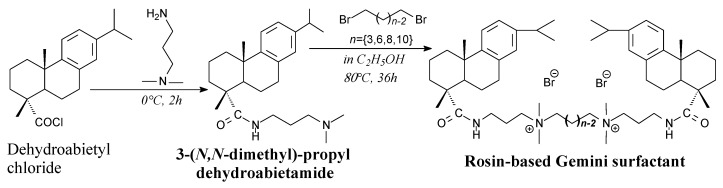
Synthesis of rosin-based gemini surfactant.

Another rosin-based gemini surfactants [143,199] can be prepared from dehydroabietyl chloride, 3-(dimethylamino)-1-propylamine and α,ω-dibromoalkanes according to Scheme 33. The product separation methods include column chromatography, vacuum drying and recrystallization from ethanol/ethyl acetate [143]. They can be used in preparation of three-dimensional mesoporous materials for separations, catalysis and drug delivery [143].

**Scheme 34:**
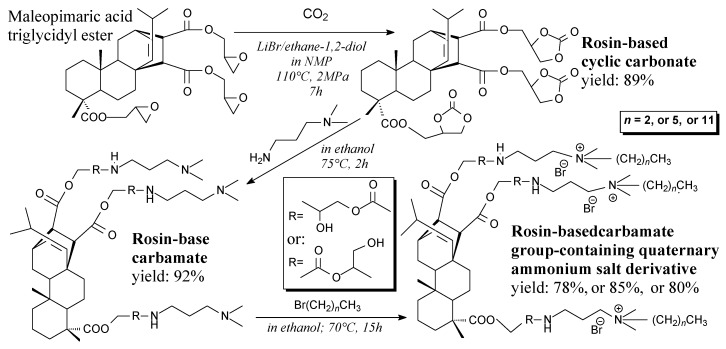
Synthesis of rosin-based carbamate and carbamate group-containing quaternary ammonium salt derivatives.

Rosin-based cyclic tricarbonate is a brown solid. It can be prepared from maleopimaric acid triglycidyl ester and carbon dioxide according to Scheme 34, prior to washing and vacuum drying [132]. Its applications include synthesis of quaternary ammonium salt derivatives [132] and non-isocyanate polyurethanes [200]. Rosin-based carbamate group-containing quaternary ammonium salt derivatives are brown solids. They can be prepared from rosin-based cyclic tricarbonate, *N,N*-dimethylaminopropylamine and alkyl bromides according to Scheme 34, and using vacuum drying and recrystallization [132]. They exhibit strong antimicrobial properties [132].

**Scheme 35:**
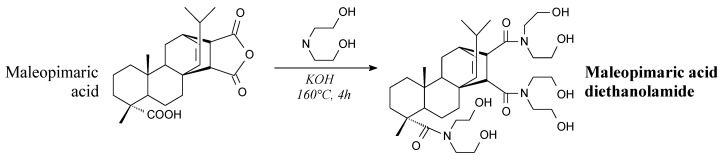
Synthesis of maleopimaric acid diethanolamide.

Maleopimaric acid diethanolamide is a solid. It can be synthesized from maleopimaric acid and diethanolamine according to Scheme 35 [103]. It can be applied as a dispersant and a viscosity depressant in coal-water slurry [103].

**Scheme 36:**
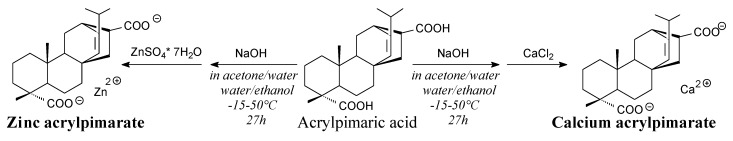
Synthesis of acrylpimaric acid divalent metal salts.

Acrylpiamric acid salts of calcium and zinc are solids. They can be prepared from acrylpimaric acid, sodium hydroxide and calcium chloride or zinc sulfate according to Scheme 36 and using such separation methods as washing, filtration and drying [137]. They can be used as a stabilizer of poly(vinyl chloride) showing better thermal stability than commercial Ca/Zn stearate [137].

**Scheme 37:**
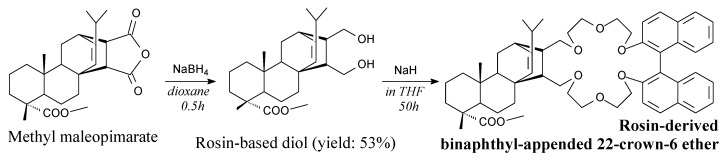
Synthesis of rosin-derived binapthyl-appended 22-crown-6 ether.

Rosin-derived binapthyl-appended 22-crown-6 ether can be synthesized from methyl maleopimarate, sodium borate and sodium hydride according to Scheme 37 [184]. It can be used in highly enantioselective reactions because of its amines enantiomeric recognition ability [184]. Very similar compounds can be also synthesized directly from maleopimaric or fumaropimaric acid [201].

#### 3.2.5. Biologically Active Compounds

Pure abietic acid isolated from rosin and its derivatives exhibit many potential activities of interest to the pharmaceutical industry, for example, antitumor, anti-inflammatory, antimycotic and anti-arteriosclerotic properties and uses in treating digestive canal, acute and chronic gastritis, and erosive gastritis, allergy, asthma, arthritis and psoriasis [202]. In this subsection rosin-based chemicals with main applications as biologically active compounds can be found. They include both medications and biocides. Their synthetic routes are usually characterized in detail and, from the chemical point of view, their TRLs are rather high. It is worth noting, that although the sustainability of their syntheses is usually worse than for other rosin derivatives (due to the necessity of using more dangerous chemicals), it is still better than their petrochemical counterparts (thanks to the use of bio-based rosin as a main substrate).

**Scheme 38:**
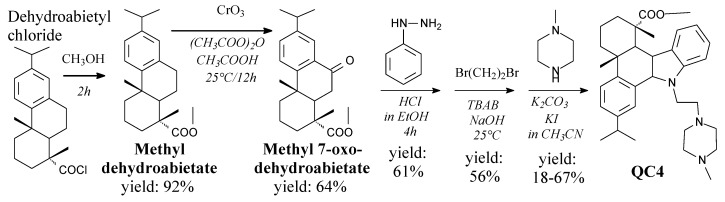
Synthesis of dehydroabietic derivative QC4.

Methyl dehydroabietate is a white solid. It can be prepared from dehydroabietyl chloride and methanol according to Scheme 38, prior to vacuum evaporation and recrystallization [141]. It can be used as a substrate in the synthesis of antimicrobial agents and medicines [141,150].

Methyl 7-oxodehydroabietate is a yellow oil. It can be prepared from methyl dehydroabietate and chromium trioxide according to Scheme 38, followed by extraction, drying and column chromatography [141]. It can be used as a substrate in synthesis of antimicrobial agents and medicines [141,150].

Dehydroabietic acid derivative QC4 can be prepared from methyl 7-oxodehydroabietate, phenylhydrazine hydrochloride, 1,2-dibromoethane and *N*-methyl piperazine, according to Scheme 38 [141,150]. It shows antimicrobial properties [150] and, moreover, induces gastric cancer cell death via oncosis and apoptosis [151]. Another dehydroabietic acid derivative QC2 is reported to be able to inhibit skin cancer cell lines [203].

**Scheme 39:**
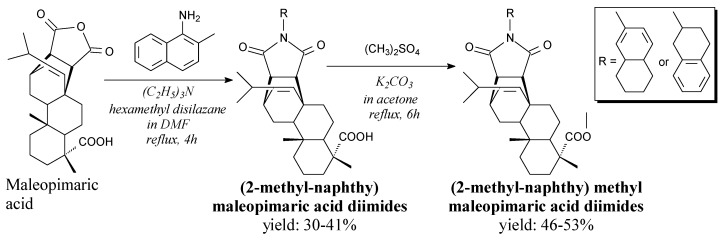
Synthesis of *N*-(2-methyl-naphthyl)maleopimaric acid diimides.

*N*-(2-methyl-naphthyl)maleopimaric acid diimides and their methyl esters are white solids (m.p. 215–290 °C). They can be synthesized from maleopimaric acid, 2-methyl-1-naphtylamine and dimethyl sulfate according to Scheme 39, and using separation methods such as extraction, washing, drying, evaporation and recrystallization [106,107]. They show significant antitumor cytotoxicity against several human cancer cell lines, especially NCI cells and MGC-803 cells [106].

Anticancer effects of various rosin derivatives, especially thioureas, were also investigated; they showed significantly cytotoxicity toward diverse human carcinoma cell lines [108].

**Scheme 40:**
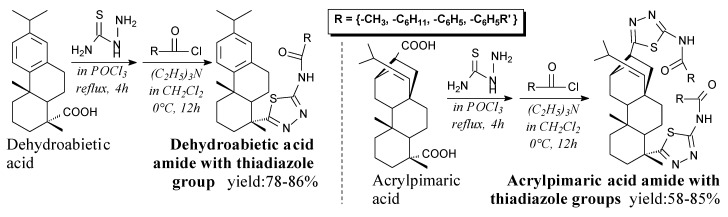
Synthesis of rosin acid amides with thiadiazole groups.

Thiadiazole group-containing amides of dehydroabietic or acrylpimaric acids can be prepared from adequate rosin acid, thiosemicarbazide and an acyl chloride according to Scheme 40, and using such separation methods as filtration, vacuum drying, recrystallization and column chromatography [130]. They can be potentially applied as insecticides [130,190].

**Scheme 41:**
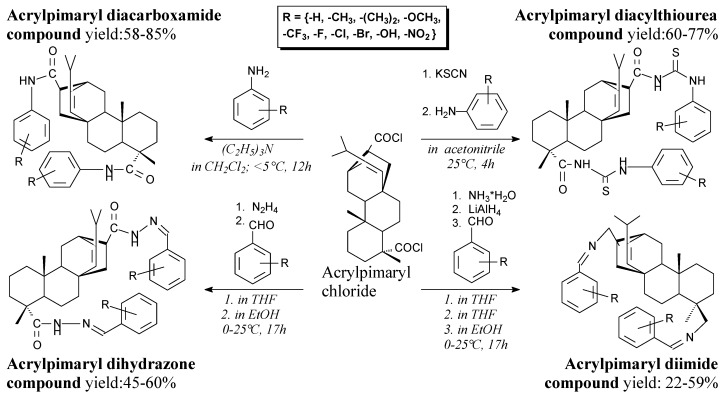
Synthesis of acrylpimaric acid-based aromatic diacylthioureas, dicarboxamides, dihydrazones and diimides.

Acrylpimaric acid-based aromatic diacylthioureas can be prepared from acrylpimaryl dichloride, potassium thiocyanate and aromatic amines according to Scheme 41, prior to vacuum evaporation and recrystallization [129]. They may be applied as botanical herbicides showing higher activity than similar dicarboxamide, dihydrazone and diimide compounds [129]. Moreover, some acrylpimaryl diimides possess antibacterial activities against Gram-positive *Staphylococcus aureus* and Gram-negative *Escherichia coli* [204]. Furthermore, some acrylpimaryl dicarboxamides show antibacterial properties against *E. coli*, whereas their activity against Gram-positive bacteria was significantly lower [205].

**Scheme 42:**
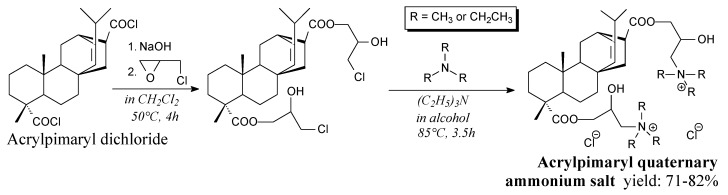
Synthesis of acrylpimaryl quaternary ammonium salts.

Solid acrylpimaryl quaternary ammonium salts can be synthesized from acrylpimaryl acid, oxalyl chloride, epichlorohydrin and volatile tertiary amines according to Scheme 41 and using such separation methods as vacuum distillation, extraction and recrystallization [189]. They show fungicidal activity and can be applied in wood preservation [189]. Very similar acrylpimaryl materials that can be applied as surfactants were also prepared [133]. Another fungicidal rosin-based materials can be prepared in similar way from rosin, epichlorohydrin and amines [134,135,136]. Maleopimaryl chloride is a yellowish solid. It can be prepared from maleopimaric acid and oxalyl (or thionyl) chloride, according to Scheme 30 or Scheme 42, prior to vacuum distillation and washing [104,105]. It can be used as an intermediate in preparation of surfactants [104] or fungicides [105].

**Scheme 43:**
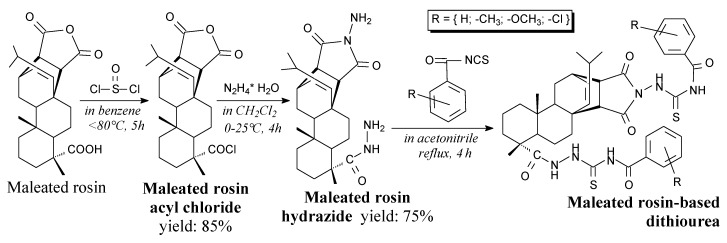
Synthesis of maleated rosin-based dithiourea compounds.

Maleated rosin-based dithiourea compounds are yellow solids (m.p. 162–216 °C). They can be prepared from maleated rosin acyl chloride, hydrazine and substituted benzoyl isothiocyanates, according to Scheme 43, and using separation methods such as rotary evaporation, recrystallization and column chromatography [105]. They can be potentially used as fungicides [105].

**Scheme 44:**
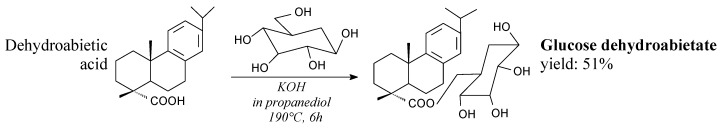
Preparation of glucose dehydroabietate.

Glucose dehydroabietate can be prepared from dehydroabietic acid and glucose according to Scheme 44 prior to vacuum distillation, filtration and washing [206]. Its potential application is as a surfactant in food industry [206].

**Scheme 45:**
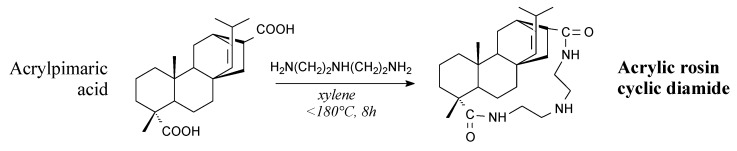
Preparation of acrylic rosin cyclic diamide.

Acrylic rosin cyclic diamide is a solid. It can be prepared from acrylpimaric acid and diethylene triamine according to Scheme 45 prior to washing and drying [131]. It can be used as a fungicide for wood preservation [131].

#### 3.2.6. Other Small/Medium Molecule Products

**Scheme 46:**
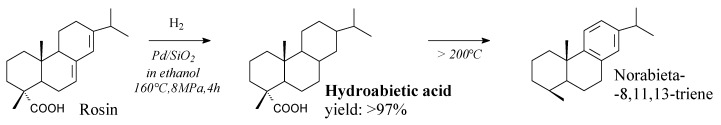
Hydrogenation of rosin to hydroabietic acid.

Hydroabietic acid can be prepared from rosin and hydrogen using palladium/SBA-15 mesoporous silica catalyst, according to Scheme 46 [207]. This catalyst is more efficient than others [208,209] Its applications include oxidation-resistant solvent-borne tackifiers and coatings, a substrate for esterification with glycerol [210], additives for polyvinylidene difluoride binders for lithium titanium oxide anodes [76]. It can be used in high-performance liquid epoxy resins [83], for synthesis of high adhesion polyurethane acrylate [173]. It is noteworthy, that non-catalytic decarboxylation of rosin can take place at temperatures above 200 °C, and the main product of such decomposition is norabieta-8,11,13-triene [211].

Rosin-based standard-quality biodiesel can be prepared in a catalyst-free process from dark-grade rosin, heavy turpentine and supercritical methanol in supercritical CO_2_ as a green medium. Yields >93% were obtained after 3 h at 340 °C, under a pressure of 11 MPa [212]. Yield of >85% can be achieved using Pt/mesoporous aluminosilicate catalyst after 4 h at 300–350 °C, 5 MPa [213]. A yield >99% can be achieved using Ni/layered double hydroxide catalyst after <2 h at 190 °C, 5 MPa [214]. Catalytic cracking of rosin is also possible to carry out using acid-activated montmorillonite [215].

**Scheme 47:**
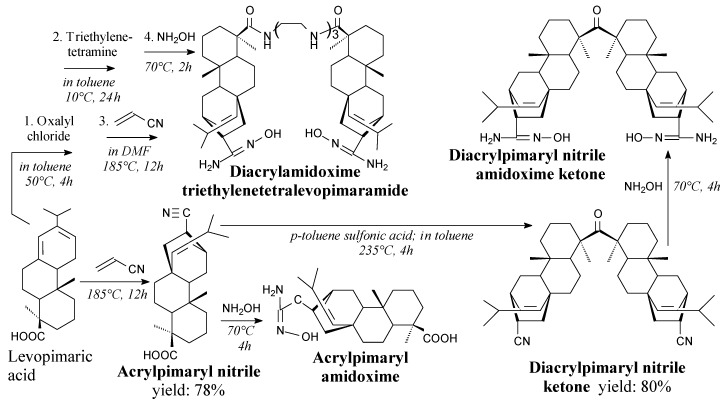
Synthesis of acrylpimaryl nitrile and acrylpimaryl amidoximes.

Acrylpimaryl nitrile can be prepared from levopimaric acid and acrylonitrile according to Scheme 47, prior to extraction, filtration and precipitation [216,217]. It can be used in synthesis of diacrylpimaryl ketone and acrylpimaryl amidoxime [217].

Acrylpimaryl nitrile amidoximes can be prepared from adequate acrylpimaryl nitriles and hydroxylamine according to Scheme 47, prior to precipitation, washing, filtration and drying [217,218]. They can be used to prepare bioactive thin films filled by magnetite nanoparticles for oil spill collecting [217] and thorium ions removal [218].

**Scheme 48:**
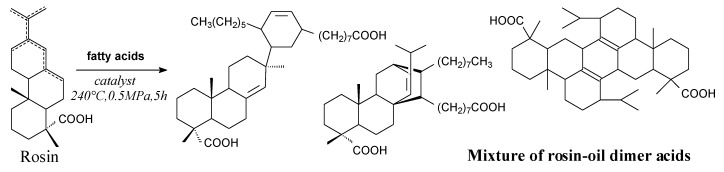
Preparation of rosin-oil dimer acids mixture.

Rosin-oil dimer acids mixture can be prepared from rosin and industrial fatty oils according to Scheme 48, prior to washing and rotary evaporation [219]. It can be used for preparation of a liquid thermal stabilizer [219]. It is noteworthy, that dimerized rosin is usually produced separately and can be applied in acrylic adhesives [220,221] to improve their wetting, adhesion and thermal stability [220].

**Scheme 49:**
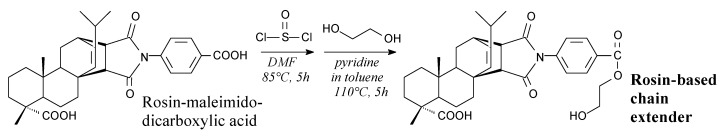
Synthesis of rosin-based chain extender for polyurethanes.

Solid rosin-based chain extender for polyurethanes can be synthesized from rosin-maleimidodicarboxylic acid, thionyl chloride and ethylene glycol, according to Scheme 49, and using such separation methods as vacuum distillation, washing and evaporation [186]. Its application in shape memory polyurethanes improves shape recovery at >1000% strain up to 96% [186].

**Scheme 50:**
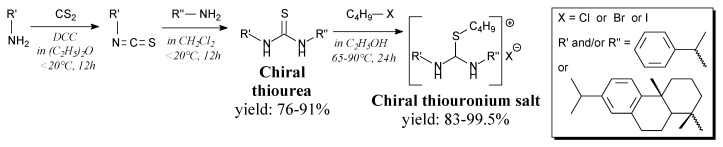
Synthesis of chiral thioureas and their thiouronium salts.

Chiral thioureas and thiouronium salts containing dehydroabietyl groups are well characterized white or yellow solids [222]. They can be prepared from chiral amines (including dehydroabietylamine), carbon disulfide and butyl halides according to Scheme 50 and using such separation methods as filtration, washing, vacuum drying, evaporation and column chromatography [222]. They can be useful for the physical separation of racemic mixtures [222]. Moreover, rosin-derived thioureas can be used as enantioselective catalysts for many reactions [12]. In recent years these were: Michael addition [223,224], tandem Michael/cyclization sequence [225], asymmetric Michael/hemiketalization [226], asymmetric aza-Henry reaction [227], asymmetric tandem reaction [228], Mannich reaction [229,230], Friedel–Crafts alkylation [231], enantio- and diastereoselective asymmetric addition [232], as well as synthesis of chiral amines [233] or *N*-protected β-amino malonates [234]. In recent studies rosin-derived thioureas are dominant majority of all investigated rosin-derived catalysts. They are not explored further in the current article because they are the subject of a comprehensive review publication [12]. For now, reports on another rosin-based catalysts are rare [235,236,237]. 

**Scheme 51:**
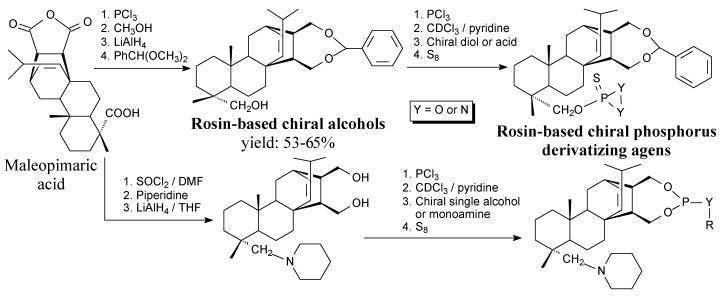
Synthetic routes of rosin-based chiral alcohols and phosphorus derivatizing agents.

Optically pure rosin-based chiral alcohols and their phosphorus derivatizing agents are white solids. They can be synthesized from maleopimaric acid in two main ways according to Scheme 51 and using such separation methods as washing, filtration, drying, evaporation, and recrystallization [113]. They can be used in ^31^P-NMR-based determination of enantiomeric excess in solutions containing chiral alcohols and amines [113].

**Scheme 52:**
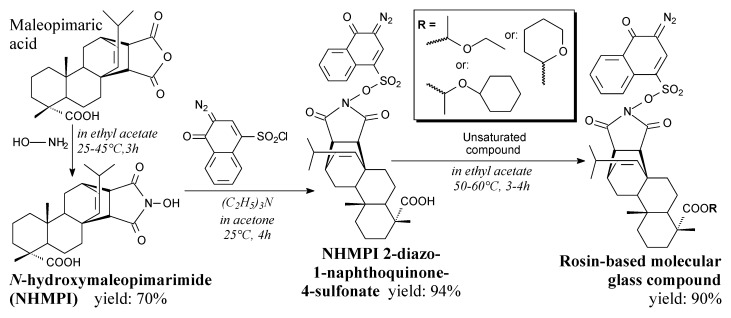
Synthesis of rosin-based molecular glass compound.

Rosin-based molecular glass photoresists can be prepared from maleopimaric acid, hydroxylamine, 2-diazo-1-naphthoquinone-4-sulfonyl chloride and unsaturated compounds: vinyl ethyl ether, or dihydropyran, or cyclohexyl vinyl ether, according to Scheme 52 and using such separation methods as filtration, washing and vacuum drying [114]. These materials can be applied in photolithography [114].

### 3.3. Macromolecular Compounds

This section describes rosin-derived macromolecular compounds with repeated units typical of macromolecules. It contains polymers, oligomers, macroinitiators and polymer functionalized materials. Additionally, some small/medium molecule compounds, which are not presented in the previous section, but necessary to obtain appropriate macromolecules, are described here. Non-toxicity, natural origin, low price, rigid structure, hydrophobicity, excellent thermal properties, anticorrosive performance, mild biocidal properties and stickiness are the most attractive reasons to use rosin derivatives in the preparation of polymer materials. A disadvantage of these processes in comparison with petrochemical counterparts is the relatively lower reactivity of rosin derivatives resulting from the steric hinderance of the diterpene skeleton and the usually lower purity of rosin-based intermediates, as discussed in Section 3.2.1. As a result, rosin-based polymer materials are usually characterized by distinct polydispersity and molecular weights rather far from their theoretical values. Therefore, their TRL is lower than for small/medium molecule compounds of rosin. It is noteworthy, that compared to non-renewable counterparts, the use of rosin significantly increases the sustainability of preparation processes according to Green Chemistry rules, so there is still high demand for basic and applied research in this field.

#### 3.3.1. Polymers for Biomedical Applications

**Scheme 53:**
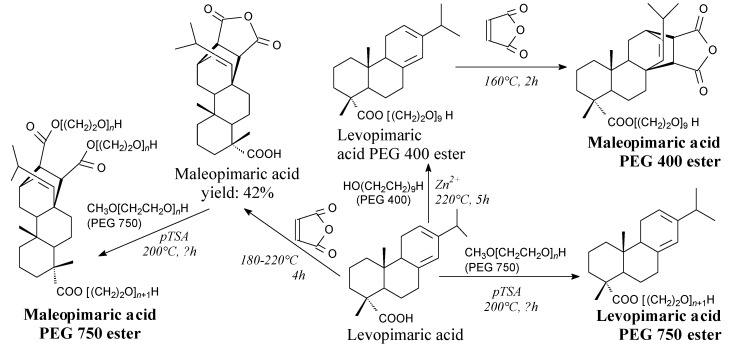
Synthesis of poly(ethylene glycol) rosin esters.

Poly(ethylene glycol) rosin esters are oligomers, which can be prepared from rosin, polyethylene glycol and maleic anhydride, according to Scheme 53 prior to drying at 40–70 °C [81,238]. Proposed applications include shells for controlled drug delivery [238,239] and dental films for periodontitis treatment [240]. Moreover, maleopimaric acid PEG esters can show carbon steel corrosion protection properties [241].

**Scheme 54:**
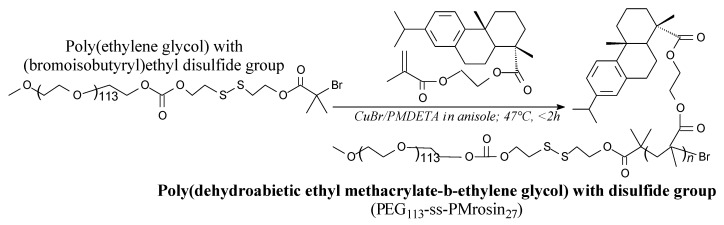
Preparation of block copolymer of dehydroabietyl ethyl methacrylate and ethylene glycol with disulfide group.

Block copolymer of dehydroabietyl ethyl methacrylate and ethylene glycol with disulfide group can be prepared via ATRP according to Scheme 54, prior to neutralization, evaporation and precipitation [168]. Potential applications include drug-delivery nanocarriers for cancer therapy [168].

**Scheme 55:**
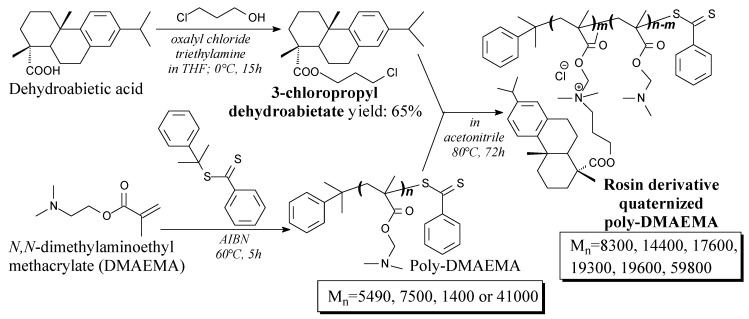
Preparation of the rosin derivative quaternized poly(dimethylaminoethyl methacrylate).

The rosin derivative quaternized poly-(*N,N*-dimethylaminoethyl methacrylate) can be prepared via “living” reversible addition-fragmentation chain-transfer polymerization (RAFT) from dehydroabietic acid, 3-chloropropanol, *N,N*-dimethylaminoethyl methacrylate and cumyl dithiobenzoate as a RAFT transfer agent, according to Scheme 55, and using gel chromatography, precipitation and vacuum drying as separation techniques [242]. It can be used as amphipathic antibacterial agent in a wide variety of biomedical and general use applications [242].

#### 3.3.2. Elastomers

**Scheme 56:**
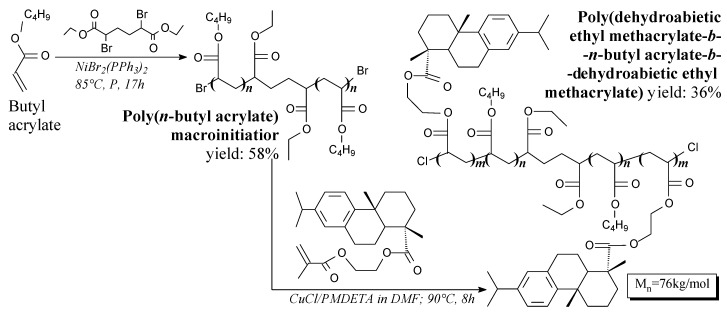
Synthesis of rosin-based triblock copolymer.

Poly(dehydroabietic ethyl methacrylate-β-*n*-butyl acrylate-β-dehydroabietic ethyl meth-acrylate) triblock copolymer can be prepared in ATRP polymerization from butyl acrylate, dehydroabietic hydroxyethyl acrylate and diethyl *meso*-2,5-dibromoadipate according to Scheme 56, prior to diluting, neutralization, evaporation, precipitation and vacuum drying [146]. Its application is sustainable thermoplastic elastomers [146].

**Scheme 57:**
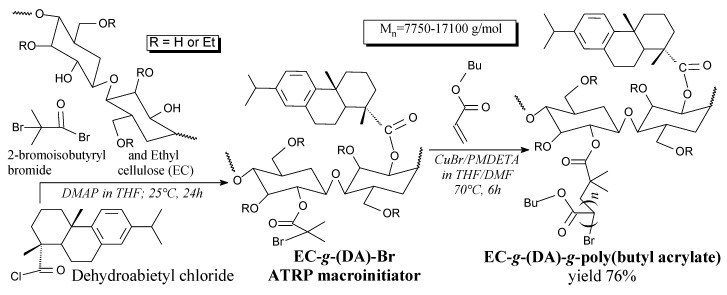
Synthesis of graft copolymer of rosin-modified ethyl cellulose and butyl acrylate.

Cellulose/rosin ATRP macroinitiators can be prepared from dehydroabietic acid, cellulose and 2-bromoisobutyryl bromide, according to Scheme 57, prior to drying at 40 °C [142]. It is used for the preparation of graft copolymers [142].

Rosin-acid-modified ethyl cellulose/butyl acrylate graft copolymer can be prepared in ATRP reaction from dehydroabietic acid, cellulose, 2-bromoisobutyryl bromide and butyl acrylate according to Scheme 57, prior to sorption of CuBr*_x_* and precipitation in methanol. [142]. Potential applications include thermoplastic elastomers and coatings with UV absorption property [142].

**Scheme 58:**
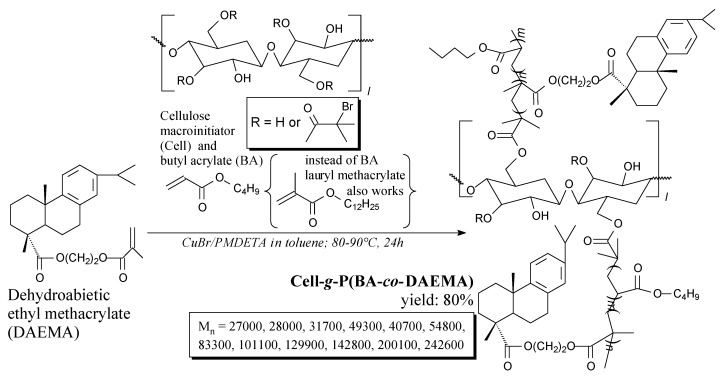
Synthesis of graft copolymer of cellulose, rosin acid and butyl acrylate (or lauryl methacrylate).

Cellulose grafted by copolymer of rosin acid ethyl methacrylate and alkyl (meth)acrylate can be prepared via ATRP, according to Scheme 58, prior to sorption and precipitation in methanol [166]. Potential applications include “green” thermoplastic elastomers having significant hydrophobic, thermal and mechanical features [166]. 

**Scheme 59:**
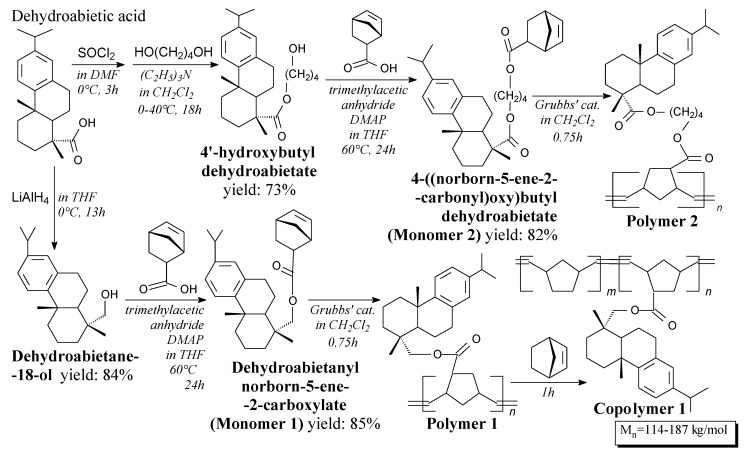
Synthesis of rosin-norbornene monomers and polymerization of them.

Rosin alcohols are colorless solids, which can be prepared in several ways according to Scheme 59, and using such separation methods like evaporation, extraction, washing and drying [243,244] [245]. They can be used in preparation of cross-linked structures with acrylamide and *N,N*′-methylenebisacrylamide [243,244], as well as norbornene-based monomers [245].

Rosin-norbornene monomers, i.e., dehydroabietanyl norborn-5-ene-2-carboxylate and 4-((norborn-5-ene-2-carbonyl)oxy)butyl dehydroabietate, are viscous oily liquids [245]. They can be synthesized from dehydroabietic acid derivatives and norbornenecarboxylic acid according to Scheme 59, prior to evaporation, washing, drying and column chromatography [245]. Its application is polymerization, or block copolymerization with norbornene, via “living” ring-opening metathesis polymerization (ROMP) process [245,246].

Homopolymers of rosin-modified norbornene can be synthesized via ROMP process according to Scheme 59 [246]. Moreover, triblock and pentablock copolymers with norbornene segments can be prepared [246]. They can be applied as bio-based thermoplastic elastomers showing well-designed architecture and high elastic recovery [245,246].

**Scheme 60:**
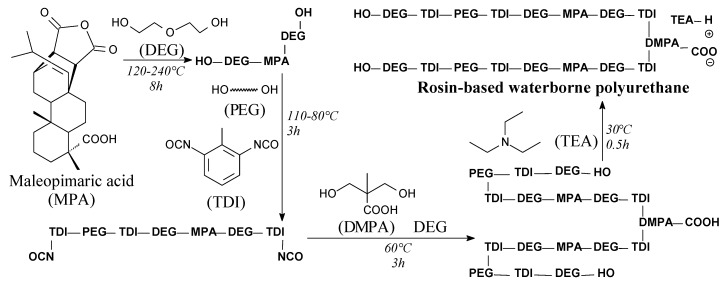
Preparation of rosin-based waterborne polyurethane.

Rosin-based waterborne polyurethanes can be prepared from maleopimaric acid, diethylene glycol, polyether glycol, toluene diisocyanate, dimethylol propionic acid and trimethylamine according to Scheme 60, and using such separation methods as vacuum drying and rotary evaporation [111]. Such polymers can be also synthesized using fumaropimaric rosin instead of maleopimaric acid [109]. These materials exhibit excellent mechanical properties, thermal stability, water resistance, antimicrobial properties against Gram-negative *Escherichia coli* and Gram-positive *Staphylococcus aureus* [109,111] and an affinity for cellulose nanocrystals [112], which allows to apply them in various biomass-based polymer and composite materials.

#### 3.3.3. Coatings and Adhesives

**Scheme 61:**
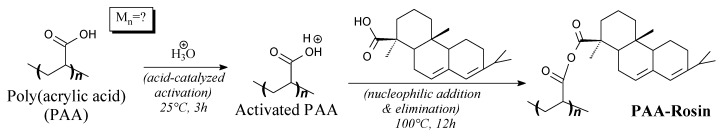
Preparation of rosin-modified poly(acrylic acid).

Rosin-modified poly(acrylic acid) is a solid. It can be prepared from poly(acrylic acid) and abietic acid according to Scheme 61, prior to washing and vacuum drying [247]. It can be applied as an excellent binder for silicon-graphite negative electrodes in lithium-ion batteries [247].

**Scheme 62:**
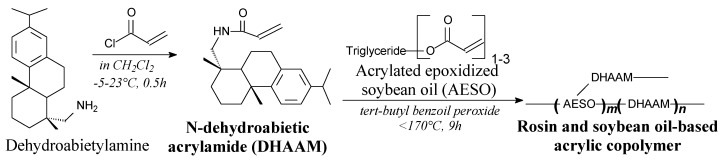
Preparation of rosin and plant oil-based acrylic copolymers.

*N*-dehydroabietic acrylamide is a white solid. It can be synthesized from dehydroabietylamine and acryloyl chloride according to Scheme 62, prior to washing and vacuum distillation. It can be used as a bio-based acrylic monomer in compolymerization processes instead of rigid petroleum-based monomers [161].

Rosin and soybean oil-based acrylic copolymers can be prepared from *N*-dehydroabietic acrylamide and acrylated epoxidized soybean oil according to Scheme 62 [161]. They are characterized by considerable hydrophobicity and heat resistivity and also other properties comparable with similar petroleum-based materials [161].

**Scheme 63:**
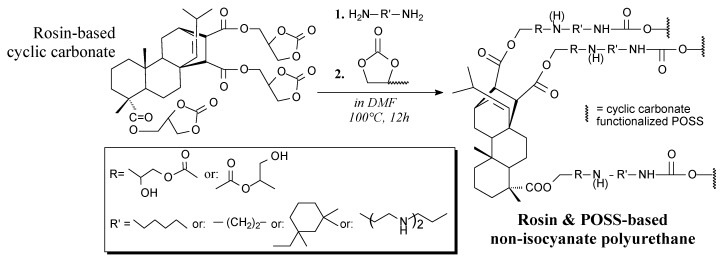
Preparation of rosin & POSS-based non-isocyanate polyurethane.

Rosin and POSS-based non-isocyanate polyurethanes can be prepared from rosin-based cyclic carbonate, polyamine and cyclic carbonate-functionalized POSS according to Scheme 63 [200]. Their applications include coatings showing improved water tolerance, hardness and thermal stability [200].

**Scheme 64:**
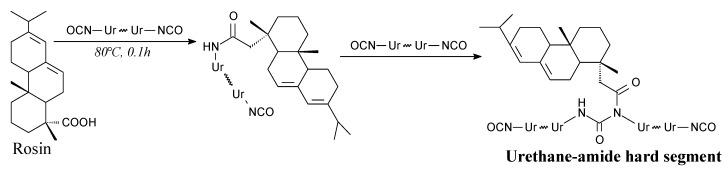
Preparation of rosin-modified urethane-amide hard segments.

Rosin can be introduced as a chain extender into polyurethanes to obtain rosin-based urethane-amide hard segments, according to Scheme 64 [248]. A potential application of the prepared materials is as sealants for non-invasive disc regeneration surgery [248]. Physical mixtures of rosin and 1,4-butanediol were also investigated for this use [249].

**Scheme 65:**
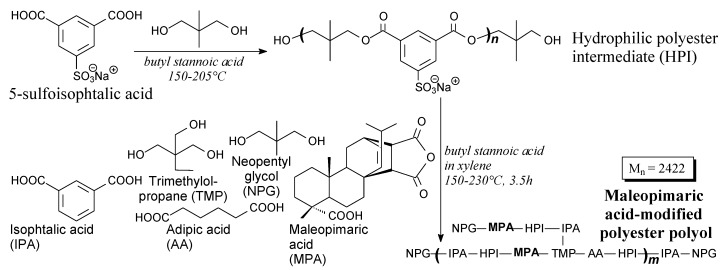
Preparation of hydrophilic maleopimaric acid-modified poliester polyol.

Maleopimaric acid-modified polyester polyol aqueous dispersion can be prepared from maleopimaric acid, adipic acid, isophtalic acid, 5-sulfoisophtalic acid, neopentyl glycol and trimethylolpropane, according to Scheme 65, prior to dissolving/dispersing in water and diethylene glycol monoethyl ether acetate as a cosolvent [91]. It can be applied in two-component waterborne polyurethane materials and coatings [110] showing improved thermal stability, hardness and resistances to ethanol and water [91,110].

#### 3.3.4. Surfactants

**Scheme 66:**
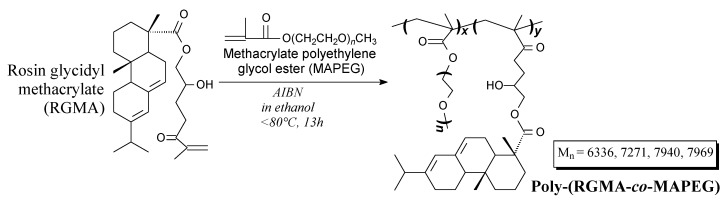
Preparation of comb-like surfactants.

Rosin-based comb-like polymeric surfactants can be prepared from rosin glycidyl methacrylate and methacrylate polyethylene glycol ester according to Scheme 66, prior to vacuum drying, precipitation, dialysis and freezing [175]. Their application include preparation of pymetrozine water suspension concentrates [175].

**Scheme 67:**
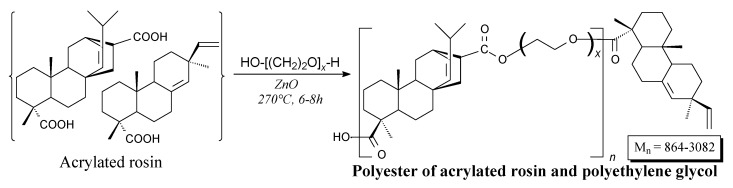
Preparation of acrylated rosin/polyethylene glycol polyester.

Polyesters of acrylated rosin and polyethylene glycols can be prepared according to Scheme 67 [125]. They can be used as surfactants in preparation of stable emulsions [125].

**Scheme 68:**
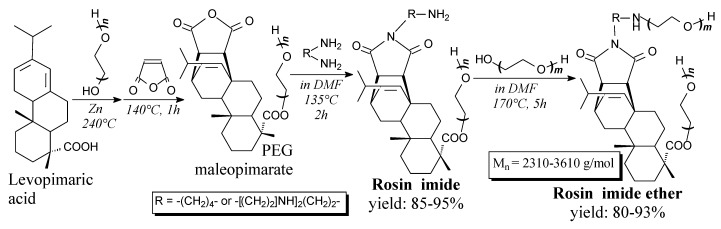
Preparation of non-ionic rosin imide polyethers.

Rosin imide polyethers are light brown solids. They can be prepared from rosin, poly(ethylene glycol) and polyamines according to Scheme 68, and using such separation methods as washing, drying, precipitation and filtration [250,251]. They can be applied as petroleum crude oil sludge dispersants [250].

#### 3.3.5. Sorbents

**Scheme 69:**
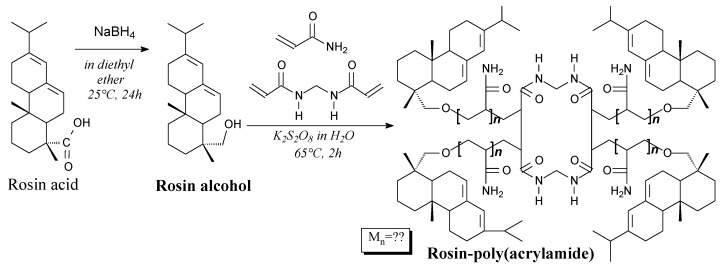
Synthesis of rosin alcohol and rosin-poly(acrylamide) star copolymer.

Rosin-poly(acrylamide) star copolymers can be prepared according to Scheme 69, prior to Soxhlet extraction using acetone and drying [243,244,252]. They can be used for wastewater treatment [243,244,252] and as a matrix for Fe_3_O_4_ nanoparticles [253].

**Scheme 70:**
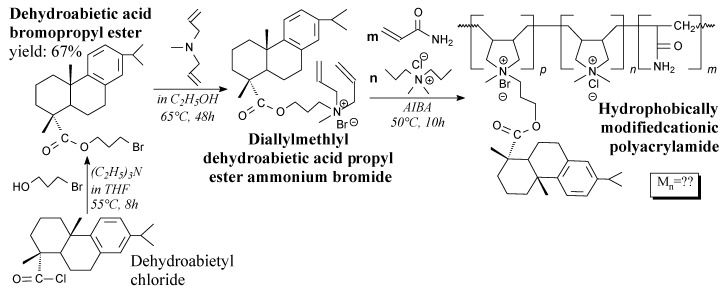
Preparation of linear rosin-poly(acrylamide) copolymer.

Linear rosin-modified cationic poly(acrylamide) can be prepared from dehydroabietyl chloride, bromopropan-1-ol, methyldiallylamine, acrylamide and diallyl dimethyl ammonium chloride in a few-step process, according to Scheme 70, followed by operations such as filtration, washing, drying and recrystallization [149]. Its utilization may be in flocculation processes [149].

**Scheme 71:**
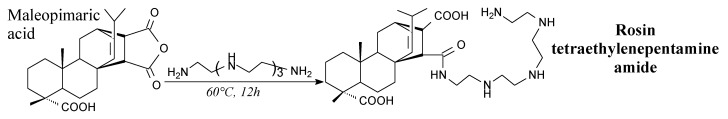
Preparation of rosin-tetraethylenepentamine amide.

Rosin tetraethylenepentamine amide is a solid. It can be prepared from maleopimaric acid and tetraethylenepentamine according to Scheme 71, prior to precipitation, washing and lyophilization [115]. It creates nano-micelles in aqueous solutions and can act as a sorbent and sinking agent in metal ions removal [115].

#### 3.3.6. Organosilicons

**Scheme 72:**
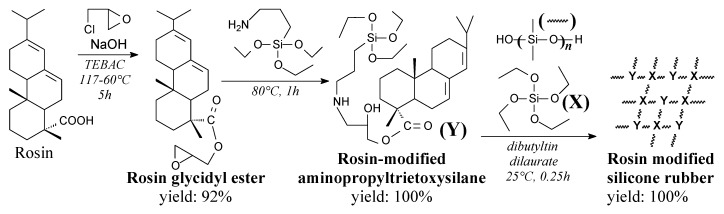
Preparation of rosin-modified room-temperature-vulcanized silicone rubber.

Rosin glycidyl ester is a brown, viscous liquid. It can be synthesized from rosin and epichlorohydrin according to Scheme 72 without further purification [254]. It can be used for preparation of cross-linking agent for silicone rubber [254].

Rosin-modified room-temperature-vulcanized silicone rubber can be prepared from rosin glycidyl ester, aminopropyltrietoxysilane, tetraetoxysilane and hydroxyl-terminated polydimetoxysilane according to Scheme 72 [254]. Rosin-modified silicones shows significantly better thermal and mechanical properties in comparison with unmodified silicone [254,255,256].

**Scheme 73:**
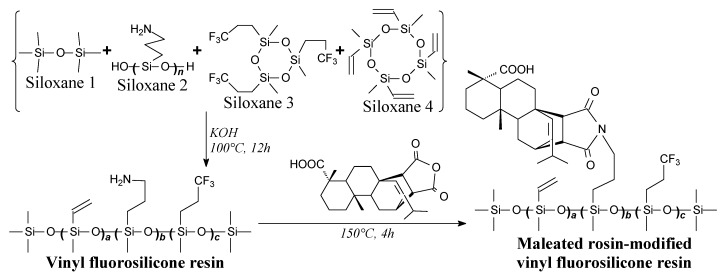
Preparation of maleated rosin-modified vinyl fluorosilicone resin.

Maleated rosin-modified vinyl fluorosilicone resin can be prepared from maleopimaric acid and siloxanes according to Scheme 73 prior to vacuum evaporation [116]. It can be used in preparation of fluorosilicone rubber, having improved mechanical and thermal properties in comparison with unmodified sample [116].

#### 3.3.7. Polysaccharides

**Scheme 74:**
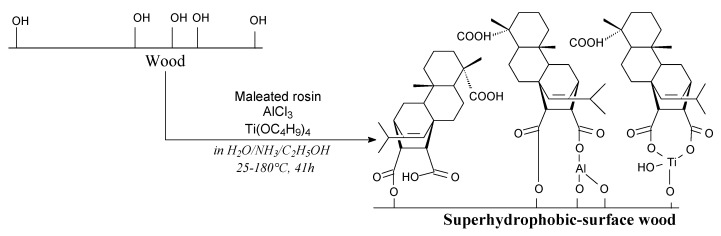
Preparation of rosin-impregnated superhydrophobic wood.

Superhydrophobic wood can be prepared using maleated rosin, aluminum chloride and tetrabutyltitanate according to Scheme 74 [119]. Such a hydrophobic material is appreciated in construction. Related starch-rosin materials have been also prepared [120].

**Scheme 75:**
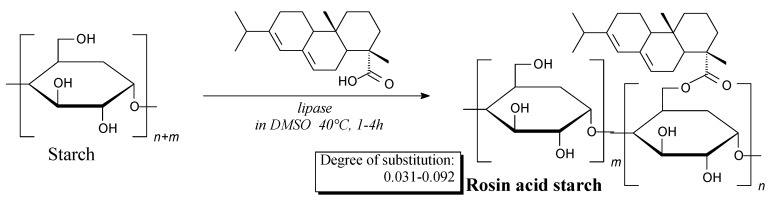
Preparation of rosin-esterified starch.

Rosin-esterified starch biopolymer can be prepared according to Scheme 75, prior to filtration, precipitation, washing and drying [257,258]. Rosin decreases solubility and swelling of starch, which potentially allows to use this polymer in food and biomedical materials [257].

**Scheme 76:**
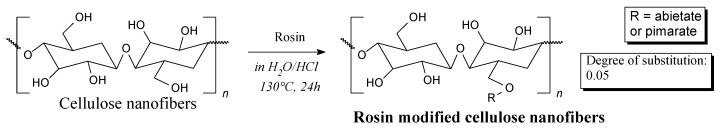
Preparation of rosin-modified cellulose nanofibers.

Cellulose nanofibers and nanocrystals surface-modified by rosin can be prepared according to Scheme 76, prior to washing in ethanol [259,260]. They can be used as antibacterial reinforcements in bio-based package films [260]. In similar way hemp fibers can be modified by tall oil rosin acids in order to improve the reinforcement adhesion to an epoxy matrix [261]. 

**Scheme 77:**
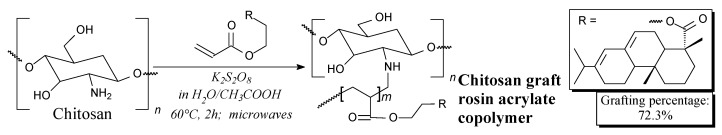
Preparation of chitosan graft rosin acrylate copolymer.

Chitosan grafted by rosin acrylate is a solid. It can be prepared from chitosan and rosin acid hydroxyethyl acrylate according to Scheme 77 prior to precipitation, filtration, washing and drying [172]. It can be potentially used in controlled release applications [172].

#### 3.3.8. Other Materials

**Scheme 78:**
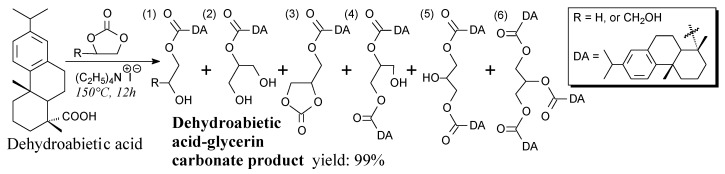
Preparation of rosin-glycerin carbonate xerographic toner.

Dehydroabietic acid-glycerol carbonate product can be prepared from dehydroabietic acid and glycerin carbonates according to Scheme 78 [262]. It can be applied in xerographic tonners [262].

**Scheme 79:**
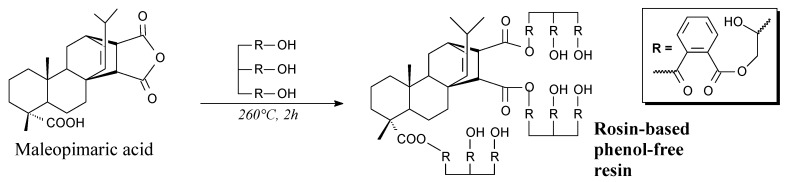
Preparation of rosin-based phenol-free resin.

Rosin-polyphthalate resin is a glossy light yellow solid. It can be prepared from maleopimaric acid, and phthalic anhydride-glycerol polyester according to Scheme 79 prior to vacuum drying [117]. Its application is an environmentally-friendly phenol-free printing ink [117].

**Scheme 80:**
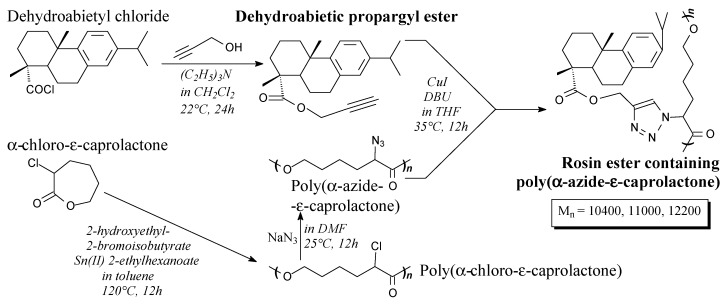
Preparation of rosin ester containing poly(α-azide-ε-caprolactone).

Rosin propargyl ester is a white, transparent liquid. It can be synthesized from rosin chloride and propargyl alcohol according to Scheme 80, prior to washing and column chromatography [147]. It can be used to prepare caprolactone graft copolymers [147].

Rosin ester containing poly(α-azide-ε-caprolactone) can be prepared from rosin propargyl ester, α-chloro-ε-caprolactone and sodium azide according to Scheme 80, and using such separation methods as centrifugation and vacuum drying [147]. This biodegradable copolymer shows properties similar to poly(methyl acrylate) [263]. Similar polymers with quaternary ammonium groups showing antimicrobial properties can be also prepared [264].

**Scheme 81:**
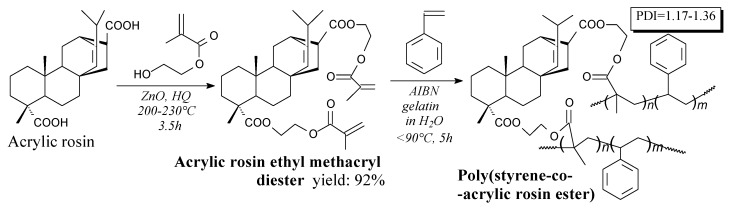
Preparation of styrene and acrylic rosin ester copolymer.

Acrylic rosin methacryl diester can be prepared from acrylic rosin and 2-hydroxyethyl methacrylate according to Scheme 81, prior to rotary evaporation and vacuum drying [124]. It can be used as acrylic monomer or resin [124].

Poly(styrene-co-acrylic rosin ester) can be prepared via suspension polymerization of acrylic rosin methacryl ester and styrene according to Scheme 81, prior to washing, filtration and vacuum drying [124]. Its application is microspheres [124].

**Scheme 82:**
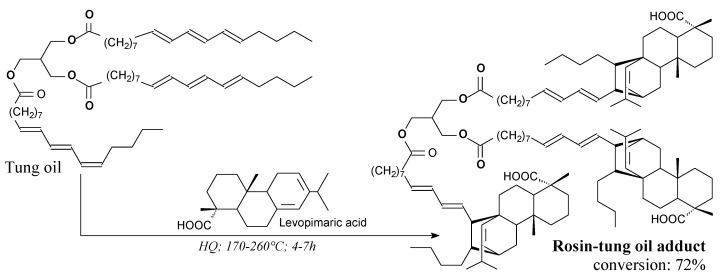
Preparation of idealized rosin-tung oil Diels-Alder adduct.

Rosin-tung oil Diels-Alder adduct is a yellowish solid. It can be prepared from levopimaric acid and tung oil according to Scheme 82, prior to precipitation and drying [265,266]. Its application is a filler, tackifier and adhesion promoter in polyurethane [265] and UV-curable adhesives [266].

**Scheme 83:**
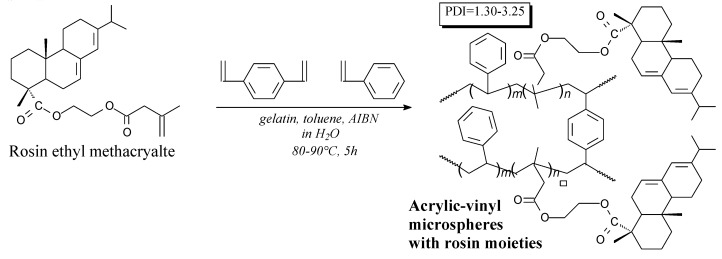
Preparation of acrylic-vinyl copolymer with rosin moieties.

Acrylic-vinyl copolymer with rosin moieties is a slightly yellow solid. It can be prepared from rosin ethyl methacrylate, styrene and divinylbenzene according to Scheme 83, prior to washing, filtration and drying [267]. Its application is polymer microspheres for adsorption and separation [267].

**Scheme 84:**
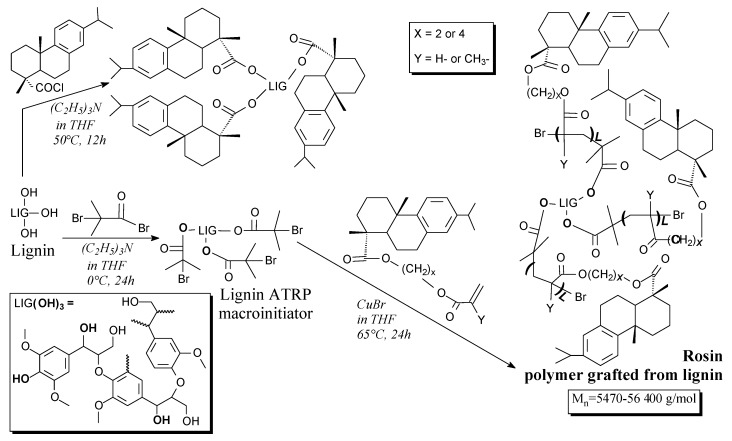
Preparation of lignin materials hydrophobized by rosin.

Rosin/lignin grafted copolymers can be prepared from lignin and dehydroabietic acid derivatives in two ways: simple grafting or ATRP polymerization, according to Scheme 84 and using such separation methods as precipitation, filtration, washing and vacuum drying [169]. These materials are characterized by strongly increased hydrophobicity compared to raw lignin [169].

**Scheme 85:**
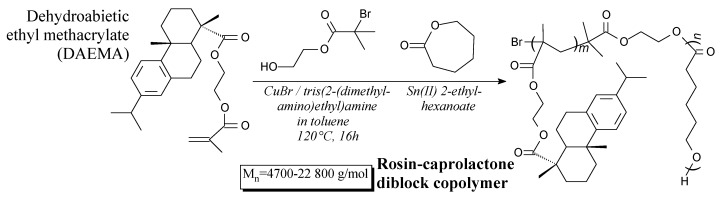
Preparation of rosin-caprolactone diblock copolymer.

Rosin-caprolactone diblock copolymers can be prepared from dehydroabietic ethyl methacrylate, ε-caprolactone and 2-hydroxyethyl 2-bromoisobutyrate according to Scheme 85 prior to precipitation and washing [170]. They can be applied in areas designed for simultaneously bio-based and biodegradable materials [170].

**Scheme 86:**
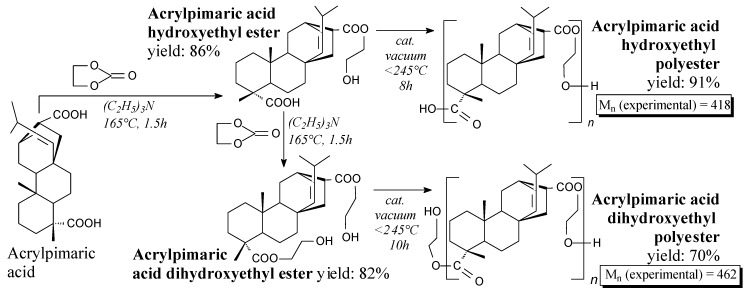
Preparation of acrylpimaric acid hydroxyethyl polyester.

Acrylpimaric acid hydroxyethyl polyesters are light yellow solids. They can be prepared via polycondensation of corresponding acrylpimaric diols according to Scheme 86 prior to grinding, washing and drying [138]. Such polymers can be potentially applied in the modern electrical and electronic industries, especially for the environmentally friendly green products [138].

Furthermore, rosin can be pyrolized in order to prepare a matrix for silver nanoparticles to apply as antibacterial filler for wooden furniture or air filter for indoors [268], a catalyst carrier with potential application as counter electrode for dye-sensitized solar cells [269], a coating for bentonite particles and support for Fe_2_O_3_ nanoparticles for chromium ions adsorption [270], and activated carbons [271,272]. 

## 4. Conclusions

Rosin is a highly modifiable raw material for both low molecular weight products and polymers. Its natural origin is accompanied with low price and a diterpene chemical nature, which is ready to introduce useful reactive chemical groups, and it exhibits a stiff constitution improving many thermal, physical, mechanical and functional properties of the final materials. In recent years rosin-based chemicals have attracted growing interest. They offer great opportunities to produce useful products: resins, curing agents, surfactants, medicines, biocides, materials for biomedical application, elastomers, coatings, adhesives, sorbents and catalysts. 

**Figure 3:**
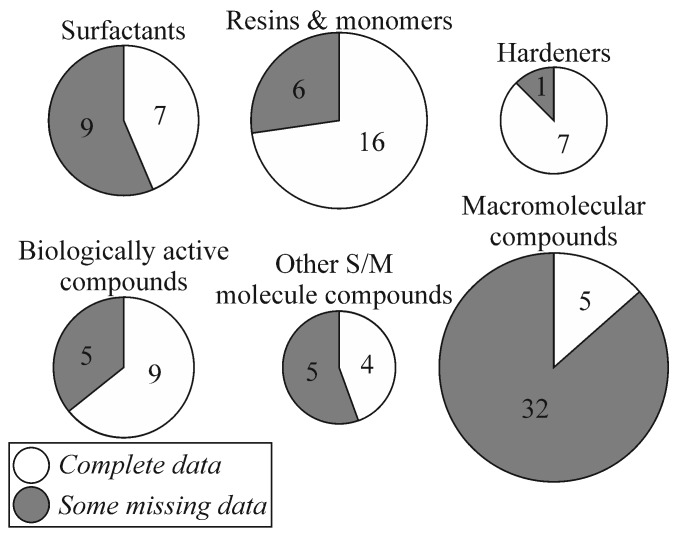
Number of complete and incomplete recipes presented in this review.

Taking into consideration the declared properties of the prepared chemicals they seem to be competitive alternatives to existing products on the market. This review presents well over 100 reproducible recipes. Sadly, some of the publications do not contain complete data, which makes it difficult to repeat the syntheses. According to our subjective opinion, the following information should be provided (or obvious) for a recipe to be considered as complete: product name, product morphology, substrate(s) names, reaction scheme, catalyst, media, temperature, pressure, time, yield and separation techniques. Figure 3 shows the number of complete and incomplete recipes in the individual parts of this review. Interestingly, the highest number of complete recipes (both absolute and relative) can be found among hardeners, resins and monomers, and also biologically active compounds. The most common missing parameter is yield. A large number of incomplete recipes in macromolecular compounds section may result from the fact that polymerizations are often carried out in order to achieve appropriate material properties, not specific yields. The summary presented in Figure 3 shows that resins, monomers, hardeners and biologically active compounds are the most completely described rosin-based chemicals in the recent decade, which makes them the most promising subjects for scaling-up and commercialization. However, this does not change the fact that rosin chemistry is able to deliver a serious amount of new environmentally-friendly solutions in many fields of science, medicine and engineering. The current review confirms this, and encourages further intensive research on rosin in the near future.

## Figures and Tables

**Table 1:** Composition of resin acids in gum rosin from different sources [23].

Species	Origin	Abietane-type Acids Content (wt.%)				Other Acids (wt.%)
		Abietic	Palustric/Levopimaric	Neoabietic	Dehydroabietic	
*Pinus massoniana*	China	39	25	16	7	13
*Pinus elliotti*	Brazil	37	15	16	5	27
*Pinus merkusii*	Indonesia	28	27	5	4	36
*Pinus sylvestris*	Russia	35	23	15	10	17
*Pinus halepensis*	Greece	45	23	13	5	14
*Pinus pinaster*	France	35	20	15	9	21
	Portugal	34	21	19	9	17
	Spain	26	22	27	6	19
	USA	14	39	18	4	25

## Abbreviations

AA	adipic acid;
AIBA	2,2′-azobis(2-methylpropionamidine) dihydrochloride;
AIBN	2,2′-azobisisobutyronitrile;
ATRP	atom transfer radical polymerization;
BA	butyl acrylate;
Bu	butyl group;
Cat.	catalyst;
DA	dehydroabietic acid;
DBU	1,8-diazabicyclo[5.4.0]undec-7-ene;
DCC	*N,N′*-dicyclohexylcarbodiimide;
DDPD	dehydroabietyl phosphate diester;
DMAP	4-dimethylaminopyridine;
DMF	dimethylformamide;
DMSO	dimethyl sulfoxide;
EC	ethyl cellulose;
EEW	epoxy equivalent weight;
Et	ethyl group;
HDMA Cl	hexadecyltrimethylammonium chloride;
HDTMAB	hexadecyl trimethyl ammonium bromide;
HPI	hydrophilic polyester intermediate;
HPLC	high-performance liquid chromatography;
HQ	hydroquinone;
IPA	isophtalic acid;
JCR	Journal Citation Reports;
m.p.	melting point;
Me	methyl group;
MEK	methyl ethyl ketone;
MPA	maleopimaric acid;
NMP	*N*-methyl pyrrolidone;
NMR	nuclear magnetic resonance;
NPG	neopentyl glycol;
PAA	poly(acrylic acid);
PEG	poly(ethylene glycol);
Ph	phenyl group;
PMDETA	pentamethyldiethylenetriamine;
PPh3	triphenylphosphine;
PSA	pressure sensitive adhesives;
pTSA	p-toluene sulfonic acid;
RAFT	reversible addition-fragmentation chain-transfer polymerization;
ROMP	ring-opening metathesis polymerization;
UV	ultraviolet;
TBA Br	tetrabutylammonium bromide;
TEBAC	benzyltriethylammonium chloride;
THF	tetrahydrofuran;
TMP	trimethylolpropane;
TPT	tetraisopropyl titanate;
TRL	technology readiness level;
